# Barriers to recruitment in a pre-surgical trial for ductal carcinoma in situ: an exploratory qualitative study of at-risk women, survivors, and providers

**DOI:** 10.1007/s10549-025-07742-2

**Published:** 2025-07-03

**Authors:** Elizabeth J. Adams, Veronica Zheng, Robin Cooper, Samantha Warwar, Denisha Brown, Lauren Schulte, Danielle Kline, Swati A. Kulkarni

**Affiliations:** 1 Northwestern University Feinberg School of Medicine, Chicago, IL USA; 2 Robin Cooper Research Group Inc, Winter Garden, FL USA; 3 Department of Surgery, Feinberg School of Medicine, Northwestern University, Chicago, IL USA; 4 Center for Health Equity Transformation, Northwestern University, Chicago, IL USA; 5 Robert H. Lurie Comprehensive Cancer Center, Northwestern University, Chicago, IL USA; 6 Division of Breast Surgery, Feinberg School of Medicine, Northwestern University, Evanston, USA

**Keywords:** DCIS focus groups, Qualitative research, Clinical trials

## Abstract

**Purpose:**

A qualitative focus group study was conducted to explore the perceived motivators and barriers to clinical trial participation from the perspective of women at risk for ductal carcinoma in situ (DCIS), women with a history of DCIS, and healthcare providers who care for patients with DCIS to improve recruitment to a prospective multicenter randomized prospective window of opportunity study of a tissue selective estrogen complex in postmenopausal women with DCIS.

**Methods:**

Qualitative thematic analysis was applied to six online focus groups. The perceived motivators and barriers to participation in DCIS presurgical trials were assessed across groups. Baseline knowledge of DCIS and attitudes toward postmenopausal hormone therapies were also evaluated. Analysis was completed in NVivo.

**Results:**

Potential DCIS pre-surgical trial participants were motivated by altruism, receiving better care, and increased monitoring by the healthcare team. Conversely, the potential for the study drug to cause harm, distrust in the medical field, and the non-life-threatening nature of DCIS were barriers to participation in pre-surgical trial. Healthcare providers felt that participants were motivated by financial incentives and receiving better care. Surgical delays, an ineffective intervention, and participant anxiety were seen as barriers to participation.

**Conclusion:**

There was overlap in the perceived motivators and barriers to participation in a pre-surgical trial among women with a history of DCIS and at-risk women. However, healthcare providers identified differing perceived patient motivators and barriers from these groups, indicating that a greater understanding among health care providers of patient motivators and barriers may facilitate recruitment to DCIS pre-surgical trials.

**Supplementary Information:**

The online version contains supplementary material available at 10.1007/s10549-025-07742-2.

## Introduction

Ductal carcinoma in situ (DCIS) is a non-obligate precursor of invasive breast cancer (IBC). Approximately 20–50% of DCIS cases originally misdiagnosed as benign lesions, and thus untreated, progressed to IBC 1–31 years later [1–3]. Controversy exists as to whether DCIS is classified as a cancer because it lacks the ability to metastasize [4]. Currently, surgery, radiotherapy, and endocrine therapy are the mainstays of DCIS treatment. DCIS has an excellent prognosis after treatment with a 3% mortality at 20 years. [5] However, DCIS is a heterogeneous disease and as our understanding of the biology of progression to IBC continues to evolve, a more tailored approach to treatment is needed.

Conducting clinical trials in newly diagnosed DCIS patients is essential to develop targeted therapies and to evaluate potential prevention interventions in high-risk women. Window of opportunity trials (WOO) are clinical trials in which participants receive a short-term intervention with the objective of measuring translational endpoints [6–8]. WOO are appealing in the presurgical setting because of the availability of the diagnostic biopsy and excised surgical tissue to measure alterations in tissue biomarkers. However, WOO trials are challenging to recruit to because patients are approached at their initial appointment when they are anxious about their diagnosis, and they are not expected to benefit from participation [7].

The impetus for the present study was to improve recruitment to a WOO entitled, “A Large-scale Multicenter Phase II Study Evaluating the Protective Effect of a Tissue Selective Estrogen Complex (TSEC) in Women with Newly Diagnosed Ductal Carcinoma in Situ,” or the PROMISE Study (NCT02694809) [9]. The main objectives of the study were to determine if taking conjugated estrogens/bazedoxifene (Duavee®) alters markers of progression in DCIS lesions. For the trial, postmenopausal women with ER + DCIS were randomized to placebo or study drug for 28 ± 7 days prior to surgery. Recruitment was completed in August 2024.

We applied qualitative research methodology to focus groups composed of postmenopausal women, women with a history of DCIS, and health care providers. The objective of this qualitative focus group study was to better understand the perceived motivators and deterrents to participating in a DCIS clinical trial from the perspectives of these groups. We also explored each group’s familiarity with DCIS and their opinions on postmenopausal hormone therapy (PHT). The findings from our exploratory focus group study are presented below and may provide insight and perspective to those planning to develop and conduct presurgical trials in patients with DCIS in the future.

## Materials and methods

A qualitative focus group study was conducted to identify factors that could influence enrollment of DCIS patients to presurgical WOO trials. Focus groups allowed us to gain insights from multiple perspectives and compare those perspectives to identify key reasons behind perceptions and corresponding behaviors [10–13]. Message laddering and brainstorming techniques were employed in each focus group [11, 13]. This study received a waiver from the Northwestern University IRB.

Insights & Outlooks, a third-party research marketing company, recruited participants from Murry Hill National, LLP’s national database. Because the PROMISE study was a multicenter trial, recruiters identified potential participants from around the United States. Potential participants were contacted, provided with a date and time for the proposed focus group, and asked if they were available and willing to be screened. Individuals who met the screening qualifications for each group were verbally consented prior to the scheduled focus group. Inclusion criteria for the participants are in Table 1.

**Table 1:** Inclusion Criteria for Participants

Focus Group	Study criteria	Focus Group Criteria
PMW	-Female -No personal or familial tie to market, pharmaceutical, or advertising -No participation in market research related to clinical trials in preceding 6 months	Postmenopausal* No history of any kind of breast cancer or precancerous condition
HxDCIS		Receive a personal diagnosis of DCIS
HCPs	-No participation in market research related to clinical trials for breast cancer in preceding 3 months	Physician, advanced practice provider, or nurse Work in oncology, breast surgery, general surgery with a focus on breast surgery, obstetrics and gynecology, or primary care setting Physicians are board certified in their specialty Care for 25 + DCIS patients (new and return) per year Care for 250 + breast cancer patients (new and return) per year Have 2–35 years of clinical experience

*Postmenopausal was defined was not having a menstrual period for 12 or more consecutive months

*PMW* Post-menopausal women focus groups

*HxDCIS* Focus group with patients who had a history of DCIS

*HCPs* Healthcare provider focus groups including physicians and nurses

The sample size to achieve saturation was determined by Outlooks & Insights based on the study goals, complexity of the topic being studied, and sampling strategy [14]. Six focus group interviews were conducted with a total of 23 participants. Three groups were composed of postmenopausal women (PMW). These women were considered at risk for developing DCIS because of their sex and menopausal status. One focus group consisted of women who had been diagnosed and treated for DCIS in the past 5 years and had not participated in clinical trials during their treatment (HxDCIS). Two groups consisted of healthcare providers (HCP)s. The characteristics of the studied groups are in Table 2.

**Table 2:** Focus Group Participant Characteristics

	PMW	HxDCIS	HCPs	
			Physician Group	Nurse Group*
# of Focus Groups	3	1	1	1
# of Participants	11	4	4	4
Sex Female Male	100.0% –	100.0% –	50.0% 50.0%	100.0% –
Age Mean (range)	56.5 (47–71)	58.3 (54–65)	–	–
Race White Black Asian Hispanic	36.4% 36.4% 0.0% 27.3%	75.0% 0.0% 0.0% 25.0%	50.0% 25.0% 25.0% 0.0%	50.0% 25.0% 25.0% 0.0%
Awareness of living a “healthy lifestyle” from 1 (not at all) to 10 (very) Mean (range)	8.2 (7–10)	8 (7–9)	–	–
Awareness of maintaining healthy diet and weight from 1 (not at all) to 10 (very) Mean (range)	8.1 (5–10)	7.5 (6–8)	–	–
Mammogram in the last 18 months	80%	100%	–	–
Regular checkup in the last 18 months	100%	100%	–	–
Years in practice Mean (range)	–	–	17.3 (14–23)	20.8 (12–29)
Practice Setting Academic Community or Private Practice	– –	– –	25.0% 75.0%	75.0% 25.0%

^*^We attempted to recruit advanced practice providers but registered nurses composed the group exclusively

Focus groups were conducted in February 2021 using the video conferencing platform Civicom®. The Civicom platform has enhanced security features and allows the facilitator to upload all materials on the website and conduct an online discussion. Each session lasted 75 min. The focus groups were moderated by author RC. RC was visible to participants and facilitated each session using the “Discussion Guide” (Supplemental Fig. 2) developed for each group by authors SK, LS, DB, and RC. The current manuscript focuses on the perceived motivators and barriers to DCIS clinical trial participation, awareness of the differences between DCIS and IBC, impressions about the time to treatment of DCIS, and perceptions about PHT. For PMW and HxDCIS, the moderator did not consistently specify the duration of the presurgical intervention or use the specific term "window of opportunity” trial, but clarified that the participant would learn about the trial after diagnosis, participate while waiting for surgery and there would be an unknown benefit to participating. The focus groups were viewed in real-time by the research team, but the team was not visible to participants. All focus group participants received compensation for their participation.

The focus groups were audio-recorded, transcribed verbatim, and uploaded into Nvivo13, a qualitative data analysis software program. Thematic analysis on the focus groups was conducted using directed content analysis [15–17]. Our team collectively defined anticipated overarching themes based on our study objectives. Each theme was transcribed into a code, or a short-hand phrase dedicated to representing a greater theme. Codes were defined in a codebook. The final iteration of the codebook is in Supplemental Table 1. Codes were used to organize and subsequently analyze the focus group transcripts by highlighting the text that pertained to that theme and to identify if text in the transcripts was related to participant knowledge of the discussion topics. Codes delineated whether the text referred to patient knowledge and beliefs, positive attitudes, negative attitudes, and neutral attitudes, as appropriate for each major theme (e.g., motivators for DCIS clinical trial participation).

The codebook was used by three independent reviewers (EJA, VZ, SW) to analyze each focus group transcript using line-by-line coding [18]. The reviewers met frequently to ensure inter-rater reliability and refine codes as needed. Discrepancies between the reviewers were resolved through discussion. Another team member, SK, was the arbiter when reviewers could not reach consensus. After completion of coding, EJA, VZ, SW, and SK compared salient concepts discovered within and between focus groups. Cohen’s kappa coefficient was 0.695 (± 0.393) for all codes related to motivators and barriers to clinical trial participation across focus groups, indicating substantial inter-rater reliability among reviewers [19]. Given the nature of focus groups, all analyses are at the focus group level rather than at the individual level.

## Results

### Participant characteristics

Characteristics of the participants are available in Table 2. All PMW and HxDCIS participants were women. The mean age was similar for all groups. The PMW focus groups self-identified evenly as White, Black, and Hispanic. For the HxDCIS group, most participants identified as White, and one-quarter identified as Hispanic. Half of the physician participants in the HCP group identified as women and all nurses in the HCP group were women. HCP participants were from a mixture of academic and community practices.

### Understanding the difference between IBC and DCIS

PMW knew that there are four stages of IBC and that higher stages are associated with worse outcomes, but none had heard of DCIS prior to participating in the focus group. Likewise, none of the HxDCIS participants had heard of DCIS prior to their diagnosis. They reported that their doctors explained the difference between IBC and DCIS well. However, HxDCIS participants did not understand the time to progression from DCIS to IBC and felt that invasion and metastasis could occur at any time. Therefore, they believed DCIS should be treated as soon as possible. One HxDCIS participant shared, *“It seemed like I had to make a decision fairly quickly as to what to do…a little bit like you’re a ticking time bomb as to when is it really going to progress into being something that isn’t manageable any longer.”* HCPs felt they explained the difference between DCIS and IBC well and that their patients understood the difference. HCPs disagreed on how swiftly DCIS should be treated and whether DCIS should be considered cancer or not. One medical oncologist shared, “*[DCIS] is not cancer. I tell them this is a premalignant condition that as long as the surgeon took out all the DCIS, we cut the risk of cancer…”* Another surgeon expressed the opposite opinion, stating, “*DCIS is a really favorable prognosis but it’s still cancer.”*

### Timing of surgery

PMW groups were informed that surgery is definitive treatment for DCIS. They unanimously stated that surgery should be done as soon as possible after diagnosis. Specifically, if surgery was scheduled more than a week after diagnosis, PMW were concerned that the DCIS could become IBC and metastasize, and their physicians could not reassure them otherwise. One PMW shared, *“If it’s cancer, it’s not going to just make one little spot of cancer and just stay like that. It’s always going to get bigger and worse.”* HxDCIS participants waited six to eight weeks between diagnosis and surgery and felt relatively assured by their physician that DCIS would not spread during this time. One participant shared, “*They said it was localized so there was no thought of it spreading.”* Another noted however*, “I would still get my panic moments where it’s like okay, he did say try really not to worry because it’s not likely to spread. We’ve got a plan.”*

HCPs reported prompt surgical treatment for their DCIS patients with an average of one to two weeks between their initial consultation and surgery. Physicians reported that their patients understood it would be highly unlikely for their DCIS to become invasive over this time, as one shared, “*Most people do understand that another month or another two weeks is not going to make a real difference in the long run.”* However, nurses reported significant patient anxiety, as one expressed, “*almost everybody is always worried*” and another shared *“[patients] sort of almost in their mind think it’s a death sentence.”*

### Postmenopausal hormone therapy

Because the PROMISE Study included a TSEC as the intervention, we wanted to understand how patients perceived PHT. PMW and HxDCIS participants had a negative perception of PHT, with concerns about developing cancer in the future. One PMW shared, “*My mom said, ‘Don’t do hormone therapy. It causes cancer,’ I don’t know if it was breast cancer, but it was a negative thing to mess with.”* PMW and HxDCIS most frequently associated PHT with breast cancer. PMW or HxDCIS did not differentiate between short-term and long-term use of PHT in terms of long-term side effects. When asked specifically if taking PHT for a few weeks would increase their risk of cancer, the majority of HxDCIS and PMW said yes. Conversely, HCP felt that PHT was safe and indicated in postmenopausal women with severe symptoms that impacted their quality of life. Some even felt a short course in a woman with a history of breast cancer could be considered if the patient had had significant symptoms. Nurses particularly felt that patients were afraid of PHT increasing the risk of IBC. One nurse shared, “*I really don’t think most of the patients understand [PHT] until the doctor explains it. Then they will research it, but I think they worry about the side-effects*.”

### Clinical trial participation

#### Perceived motivators

**Fig. 1:**
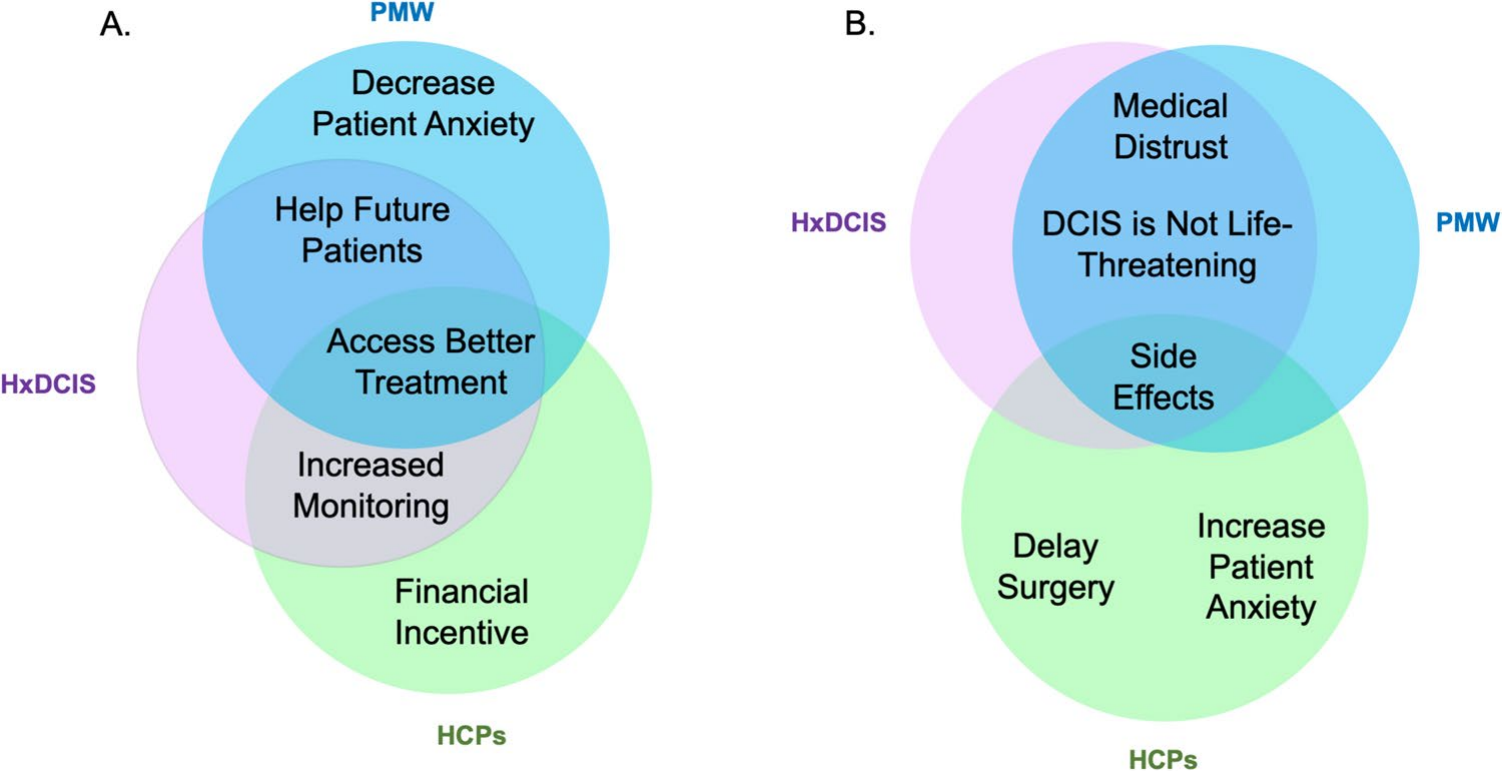
Similarities and differences among top perceived motivators (**A**) and barriers (**B**) to DCIS clinical trial participation. PMW focus groups (blue). HxDCIS focus group (pink). HCPs focus groups (green)

All participants were asked what they believed would motivate newly diagnosed patients with DCIS to participate in a clinical trial. Figure 1 depicts the similarities and differences among each group’s top three themes when discussing perceived motivators for clinical trial participation. PMW identified: 1.) helping future DCIS patients; 2.) accessing better treatment; and 3.) reducing anxiety associated with their diagnosis as their most frequently identified motivators. The DCIS group believed: 1.) accessing better treatment; 2.) increased monitoring by the medical team; and 3.) helping future DCIS patients were the most important motivators for participation. HCPs perceived the top motivators as 1.) increased monitoring by the medical team; 2.) financial compensation; and 3.) access to better treatments. Representative quotes of each of the top themes by focus group type are in Table 3.

**Table 3:** Top themes and representative quotes for perceived patient motivators to DCIS clinical trial participation

Group	Theme	Representative Quote
*Motivators*		
PMW	*Help future DCIS patients*	“I’m now 60, and a lot of the medicines that I am now taking, for example for high blood pressure or any other thing that I have, I’m assuming somebody before me, maybe in their 80 s right now, was a part of some kind of a study, so that I could benefit later. It’s like…paying it forward”
	*Access better treatment*	“Maybe it might be a cure or maybe she might get better under a clinical trial.”
	*Ease anxiety*	“Joining could relieve her anxiety, thinking that what the doctor is doing might not be enough, so [participating in the trial would be going] the extra mile.”
HxDCIS	*Access better treatment*	“Have access to something that’s very likely to be helpful but you can’t get it as a general public. […] A lot of times, it seems these trials I had were medications or things they have are super good and super helpful.”
	*Increased monitoring by healthcare team*	“Getting more frequent health checkups as part of the clinical trial because I would presume if I’m going to do clinical trial that they’re going to keep a good tab on me and that that so I would be getting more frequent health checkups which would be a good thing to be watched. You’re under constant supervision, constant care.”
	*Help future DCIS patients*	“I think the idea that you could be helping to get a good drug approved, helping science – helping the cause, so to speak, is a positive association in my mind. They need real patients. Without real patients with real diagnosis, they wouldn’t be able to do these studies. That would be one of the biggest positives to me too, do what I could to help further the cause and treatment of breast cancer.”
HCPs	*Increased monitoring by healthcare team*	“I always tell my patients when they participate in clinical trials, they actually get better monitoring by the nurses, by the doctors, and by the clinical trial coordinators. So, everything is provided at the exact time. There’ll be no delays, there’ll be no exceptions, they’ll be very well taken care of. So, I think that’s also comforting to know for the patients.” [Physician] “There’s med students that are involved, interns, residents, the whole thing. So I think that the more eyes on someone, the better off they are. That they’re seeing, are we seeing something good, are we using some side-effects that we need to hold back on?” [Nurse]
	*Financial incentive*	“Sometimes the trials help some assistance and offer care, you know, some aid. I think that cost might be an issue. They may get free treatment or low-cost treatment.” [Physician] “I think a lot of us, we do okay as nurses, but certainly wouldn’t mind some extra money. I think it’s a motivator.” [Nurse]
	*Access better treatment*	“I think also that it’s very possible that the current clinical trial therapy could be more effective than the standard of care. So, she basically is getting a therapy ahead of everybody else” [Physician] “They’re being offered something that others aren’t.” [Nurse]

*PMW* Post-menopausal women focus groups

*HxDCIS* Focus group with patients who had a history of DCIS

*HCPs* Healthcare provider focus groups including physicians and nurses

#### Perceived deterrents

All focus groups were asked what they believed would prevent a newly diagnosed DCIS patient from participating in a clinical trial. The top three themes from the PMW, HxDCIS, and HCPs groups are illustrated in Fig. 1. PMW identified: 1.) potential side effects; 2.) medical distrust including concerns about receiving placebo without standard of care treatment; and 3.) the non-life-threatening nature of DCIS. The HxDCIS focus group reported the same themes as PMW but in a different order of importance. HCPs believed that barriers included 1.) delaying surgery; 2.) potential side effects; and 3.) exacerbating patient anxiety. Table 4 includes representative quotes for the most salient barrier themes by focus group type (Table 5).

**Table 4:** Top themes and representative quotes for perceived patient-related barriers to DCIS clinical trial participation

Group	Theme	Representative Quote
*Barriers*		
PMW	*Side effects*	“If it’s a trial, that means it’s not tested, you don’t know how you’re going to react, what if you have terrible side effects, what if there’s long-term damage God forbid from something in the drug that’s reacting with your particular individual body chemistry? So you’re taking a chance.”
	*Distrust of medicine*	“In the past, [African Americans] have…been given things that were not exactly positive so that’s still at the back of their minds.” “When I think of clinical trials, I think of you being the first person that they would try this medication on. The scapegoat, if I may, and I’ve also heard that there is placebo, where some people would get the real medication and some wouldn’t, and then they would make comparisons to see how that medication works. If it’s life-threatening and you would like to seek the treatment, it would be negative for me [to get placebo].”
	*DCIS is not life-threatening*	“If it’s precancerous, why are you putting something in your body? You don’t know the effects of—people administering it don’t know either that’s why it’s called a trial. So, why would you even do that to yourself?”
HxDCIS	*Side effects*	“You think of things like what if I find out years from now that maybe it helped my breast cancer but it caused another kind of cancer or it caused this or it caused that. Now, in the process of testing on me, we found out that this isn’t a helpful drug at all and now, you’ve got some sort of lingering effect.”
	*DCIS is not life-threatening*	“Clinical trials would be a good option because you run out of options but …she might be able to treat DCIS more quickly and effectively [outside of a clinical trial].”
	*Distrust of Medicine/ Receiving placebo*	“The possibility of maybe feeling like a guinea pig and the possibility of not getting—I think of a clinical trial of some people get the real thing, other people get a placebo. Well, when we’re talking cancer, I don’t want a placebo.”
HCPs	*Delays in surgery*	“The negative would be that clinical trials take a long time. So it may delay their treatment. And they also it may take a long time to get the results and the outcomes of the trial.” [Nurse] “[It could cause] some delay of so-called standard of care treatment such as surgery” [Physician]
	*Side effects*	“…You’re dealing with a novel agent and certainly, the benefits versus the risks are not that well studied yet, i.e., that’s why the drug’s being trialed.” [Physician] “…There’s additional tests required or additional biopsy required because the clinical trial requires that. So, that leads that there’s some potential complications from all the additional tests.” [Physician]
	*Increase anxiety*	“…there’s a lot of patients that hear the word ‘clinical trial”…the next thing they think is, ‘I have something. I’m dying, there’s no treatment, and now I’m in a clinical trial…There’s something they’re not telling me.’ So, it might be the psychological effect…” [Physician]
PMW: Post-menopausal women focus groups HxDCIS: Focus group with patients who had a history of DCIS HCPs: Healthcare provider focus groups including physicians and nurses		

*PMW* Post-menopausal women focus groups

*HxDCIS* Focus group with patients who had a history of DCIS

*HCPs* Healthcare provider focus groups including physicians and nurses

**Table 5:** Perceived Impact of the COVID-19 Pandemic on Clinical Trials

	PMW and HxDCIS Groups	HCP Groups
Preference to first learn/teach about one’s eligibility for clinical trials during the COVID-19 pandemic	Via written communication (e.g., email, patient portal, or text)	In-person
Comfort to be recruited/recruit for clinical trials during the COVID-19 pandemic	Comfortable discussing - Via Telemedicine - In-Person Without strong preference	Comfortable discussing - Via Telemedicine - In-Person With strong preference for in-person
Whether COVID-19 was a perceived barrier to clinical trial participation with reasons	No -Were still going to doctor anyway -Comfortable doing telemedicine for research too	Yes -Difficult to explain trial design over telemedicine -Additional visits and exposures unappealing to patients

PMW and HxDCIS participants were concerned that they would not receive standard treatment if assigned to the control arm of a trial. When the moderator clarified that they would receive standard of care after completing the trial, PMW and HxDCIS were still skeptical. PMW and HxDCIS also emphasized the excellent prognosis of DCIS and the availability of safe and effective standard treatments. Regarding the impact of the study intervention, HCPs discussed the potential that the study intervention may be ineffective in performing as it was hypothesized. Notably, HxDCIS and PMW groups perceived that study drugs in clinical trials offering financial compensation were not “as good” as study drugs in clinical trials that did not provide compensation. They felt that participants may not be as forthcoming about reporting side effects or may inflate symptom improvement if they were receiving compensation.

HCP felt that participating in a presurgical trial would increase patient anxiety by making them feel like they had a worse prognosis or that it would be an additional burden during an already overwhelming time. HCP also mentioned trying to predict which patients would be willing to participate in clinical trials based on their anxiety level before broaching the topic. This contrasted with PMW and HxDCIS, who felt that participating in a clinical trial would reduce their anxiety and make them feel that they were being proactive about their treatment. In particular, the HxDCIS group said they would feel better if they participated in a clinical trial and were not “just waiting” for their surgery. Neither PMW nor HxDCIS groups emphasized time commitment as a deterrent for participation. While HCP considered delaying surgery to be the top barrier for participation in presurgical clinical trials, PMW and HxDCIS groups did not identify this as one of their top three barriers for clinical trial participation despite wanting to proceed to surgery as soon as possible after diagnosis.

## Discussion

**Fig. 2:**
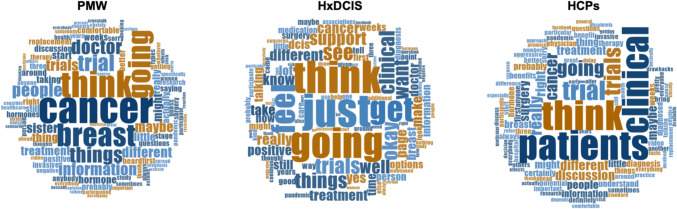
Word clouds of the one hundred most frequently used words in each of the PMW, HxDCIS, and HCP focus groups. The greater font size of the word corresponds to greater frequency of the word during the focus group. PMW, HxDCIS, and HCPs greatly varied in terms of the most frequently used words, demonstrating the heterogeneity between groups. Most strikingly, the word “cancer” was the most used word among participants in the PMW groups, while it was used the least among HxDCIS. HCPs most often utilized words focused on patient care including “clinical,” “trial,” “surgery,” and “treatment.”

Little has been published on the motivators and barriers of clinical trial participation in women with newly diagnosed DCIS. Only one randomized control trial in DCIS patients has been conducted to evaluate the impact of a decision aid on recruitment [20, 21]. To our knowledge, this is the first qualitative study to describe factors that could influence participation in a DCIS pre-surgical trial from the perspective of patients and providers (Fig. 2). The main findings were 1.) PMW and HxDCIS predicted different motivators and barriers to DCIS clinical trial participation than HCP; 2.) PMW and HxDCIS did not differentiate between DCIS and IBC, and this strongly influenced their desire to proceed with surgery immediately; and 3.) PMW and HxDCIS felt that even a short duration of PTH could lead to long-term complications.

Overall, both PMW and HxDCIS groups felt there was a sense of urgency to undergo surgery as soon as possible because they feared rapid progression to invasion and development of distant metastasis. Overestimation of risk and confusion about the diagnosis of DCIS has been previously reported [22, 23]. As demonstrated in our HCP group, this confusion is likely due in part to the lack of consistent terminology used by physicians and different perceptions about DCIS used among HCPs [24]. There is significant opportunity for clinicians to highlight that the time of progression to invasion is measured in years, not weeks, or days, and that delaying surgery to participate in a presurgical trial does not impact outcomes [2, 25]. The publication of an active monitoring trial in DCIS may reduce anxiety among patients and provides additional information for clinicians about the safety of delaying surgery for short duration [26]. For the PROMISE study, we created a DCIS information sheet for potential participants that highlighted the slow rate of progression to invasion, excellent survival outcomes, and provided them with a link to the Susan Love Video on DCIS [27].

Misinformation about PHT is prevalent among women and the side effects of PHT have been misrepresented and oversimplified for years [28, 29]. A renewed interest in safe and effective treatments for menopause may begin to reduce the stigma of PHT among women going forward. For the PROMISE study, overcoming this barrier was the greatest challenge for recruitment. We attempted to address common misconceptions about PHT in our patient pamphlet, including recent findings from the Women’s Health Initiative. We also created a PROMISE study video that summarized study aims, participation requirements, and provided information about the study drug that the patients watched as part of the recruitment process. Once we understood that patients feared long-term consequences, we directly addressed this concern as part of our approach to the patient with mixed success.

PMW and HxDCIS identified the ability to help future patients diagnosed with DCIS as a strong motivator for participation. Altruism was the top motivation for participating in clinical trials for PMW. PMW and HxDCIS acknowledged that all medications and interventions, including those that they currently use, were once studied in trials that required study participants. Previous studies have reported similar findings among early-stage breast cancer patients and found that the primary motivation to participate in clinical trials varied by type of study (i.e., Phase 1 vs. Phase 3) [7, 30]. Parikh et al. identified comparable positive attitudes toward WOO participation in patients with stage 1 and 2 breast cancer [7]. Interestingly, when HCPs were told that women in the PMW and HxDCIS groups identified helping future DCIS patients as a strong motivator, HCPs reported that they did not consider helping others to be enough of a reason for patients to participate in a clinical trial. As part of our recruitment approach, we incorporated the potential benefits to future women, including identifying a new adjuvant therapy for DCIS with fewer side effects, and identifying a treatment for menopausal symptoms in breast cancer survivors.

A major barrier identified by the PMW and HxDCIS groups was that DCIS has a favorable prognosis and effective treatment options [5, 31]. This was consistent with patients’ view that clinical trials are valid options when standard of care treatments have been exhausted. For randomized WOO trials, the potential to receive a placebo instead of the intervention was a barrier to participation for PMW and HxDCIS groups. Other qualitative studies have described similar concerns among trial participants [7, 32]. We stressed it is likely that neither group will have a clinical benefit, but important information would be gained from both the intervention and placebo arms. Finally, because the HCP group described attempting to predict patient interest in clinical trials based on stress or anxiety, we approached all patients regardless of whether we thought they would participate or not. Unger and colleagues found that lack of opportunity to participate, not patient-driven factors, was the largest barrier to trial recruitment [33].

There were differences between the HCP and PMW/HxDCIS groups regarding their perceptions of financial compensation for trial participation. PMW and HxDCIS groups viewed clinical trials that provided financial compensation with suspicion. In contrast, HCPs felt that financial compensation would be a motivator. Our findings in PMW and HxDCIS participants contrast with previously reported perceptions of potential trial participants, which found that financial compensation facilitates clinical trial recruitment because it acknowledges the patients’ time and effort, and improves retention [34]. Based on our focus group findings, we de-emphasized the financial compensation in recruitment materials and specified that any financial remuneration is intended to compensate participants for their time and incidental travel costs.

Our study has several limitations. Our study was exploratory in nature. The goal of conducting these focus groups was to learn about newly diagnosed DCIS patients’ perceptions about presurgical clinical trial participation specifically to improve recruitment to the PROMISE study, so our findings might not translate to all pre-surgical DCIS trials. Also, the qualitative nature of this analysis and the limited size of each focus group could limit the generalizability of our findings. Lastly, all study participants chose to participate in this study, thus they may favor research participation more than the general population.

## Conclusion

Our study demonstrated that PMW and HxDCIS groups reported similar perceived motivations and barriers to clinical trial participation, which differed from those of HCPs. Findings from our focus groups may provide potential opportunities for HCPs and clinical researchers to improve clinical trial recruitment in patients with newly diagnosed DCIS.

## Supplementary Information

Below is the link to the electronic supplementary material.

Supplementary file1 (DOCX 3443 KB)

## Data Availability

The de-identified transcripts and key themes from the focus groups can be accessed by contacting the corresponding author.

## Abbreviations

IBC: Invasive breast cancer
DCIS: Ductal carcinoma in situ
PTH: Postmenopausal hormone therapy
PMW: Postmenopausal women
HxDCIS: Women with a history of DCIS
HCP: Health care providers
