# Supplementary material for: Barriers to recruitment in a pre-surgical trial for ductal carcinoma in situ: an exploratory qualitative study of at-risk women, survivors, and providers
Source: Breast Cancer Res Treat. 2025 Jul 3;213(1):33–42. doi: 10.1007/s10549-025-07742-2 (PMC12259734; doi:10.1007/s10549-025-07742-2)

**Supplemental Table 1:** Focus Group Thematic Analysis Codebook

| Knowledge of DCIS | Comments from participants indicating their understanding, familiarity, and prior experiences surrounding DCIS |
| --- | --- |
| Present | Comments from participants indicating they know of DCIS and/or possess factual knowledge about DCIS (stage, prognosis, prevention, screening, treatment, etc.) |
| Not Present | Comments from participants indicating they do not know of DCIS and/or do not possess factual knowledge about DCIS (stage, prognosis, prevention, screening, treatment, etc.) |
| Knowledge of Different Types of Breast Cancer | Comments from participants indicating their understanding, familiarity, and prior experiences surrounding invasive breast cancer |
| Present | Comments from participants indicating they know of IBC and/or possess factual knowledge about DCIS (stage, prognosis, prevention, screening, treatment, etc.) |
| Not Present | Comments from participants indicating they do not know of IBC and/or possess factual knowledge about DCIS (stage, prognosis, prevention, screening, treatment, etc.) |
| Sources of Information Related to cancer, DCIS, IBC | Comments from participants about where they have gathered or would gather information surrounding cancer, DCIS, and/or IBC. This may include information regarding prevention, detection, treatment, prognosis, clinical trials, or any other information used to form perceptions about or make decisions surrounding cancer/DCIS/IBC. |
| Healthcare Providers (HCPs) | Comments from participants indicating they would seek or have sought the insight of their primary care providers, specialists, or any other healthcare provider. |
| Internet | Comments from participants indicating they would seek or have sought information from the internet, such as from Google, WebMD, informational webpages from medical centers, overseeing medical bodies such as the American Cancer Society, a drug company website, etc. Also includes social media or webpages where patients and/or providers may interphase via online discussion boards and similar platforms. |
| Friends, Family | Comments from participants indicating they would seek or have sought the insight of their friends, family, colleagues, and acquaintances. |
| Books | Comments from participants indicating they would seek or have sought information from books. |
| Informational Materials such as flyers, brochures, pamphlets | Comments from participants indicating they would seek or have sought information from informational flyers, brochures, pamphlets. Often these informational materials may be encountered in medical offices. |
| Other | Comments from participants indicating they would seek or have sought information from sources not specifically included above. Examples include fundraising and nonprofit events, fundraisers, etc. |
| Knowledge of Risk Reduction & Prevention vs. Early Detection | Comments from participants indicating their understanding, familiarity, and prior experience with risk reduction, prevention, and or/early detection of DCIS or IBC. |
| Present | Comments from participants indicating they know of the similarities and differences between prevention and early detection/screening of DCIS/IBC. |
| Not Present | Comments from participants indicating they do not know of the similarities and differences between prevention and early detection/screening of DCIS/IBC. |
| Scheduling Surgery for DCIS | Comments from participants surrounding beliefs, attitudes, preferences, risks, and benefits of various timelines for scheduling surgery for DCIS. |
| Preference surrounding time between dx and surgery | Comments from participants indicating their preferred timeline for scheduling DCIS surgery after initial diagnosis. |
| Attitudes surrounding time between dx and surgery | Comments from participants indicating their emotions, attitudes, concerns, and perceptions for scheduling DCIS surgery after initial diagnosis. |
| Positive Influential Factors for Participating in Clinical Trials specifically for DCIS | Comments from participants indicating their willingness to participate in a DCIS clinical trial. |
| Access to newer or better DCIS treatments | Comments from participants indicating that the prospect of newer or better DCIS treatments potentially offered via clinical trials is desirable and/or would positively influence their participation in a DCIS clinical trial. |
| Increased monitoring by healthcare team | Comments from participants indicating that the prospect of increased monitoring by a healthcare team potentially offered via clinical trials is desirable and/or would positively influence their participation in a DCIS clinical trial. |
| Financial incentive | Comments from participants indicating that financial incentive potentially offered via clinical trials is desirable and/or would positively influence their participation in a DCIS clinical trial. Financial incentive may include but is not limited to decreased cost of care, decreased cost of treatment, and direct compensation for participation. |
| Contributing to Science/Research | Comments from participants indicating that the prospect of contributing to science and/or research potentially offered via clinical trials is desirable and/or would positively influence their participation in a DCIS clinical trial. |
| Helping patients with the same disease in the future | Comments from participants indicating that the prospect helping future DCIS patients potentially offered via clinical trials is desirable and/or would positively influence their participation in a DCIS clinical trial. |
| All other treatment options exhausted | Comments from participants indicating that the prospect of having an additional line of treatment when all others were exhausted potentially offered via clinical trials is desirable and/or would positively influence their participation in a DCIS clinical trial. |
| Other | Comments from participants indicating that the prospect of another influential factor not specifically listed above is potentially offered via clinical trials and is desirable and/or would positively influence their participation in a DCIS clinical trial. For example, this may include having a family history of breast cancer. |
| Negative Influential Factors for Participating in Clinical Trials specifically for DCIS | Comments from participants indicating their lack of willingness to participate in a DCIS clinical trial. |
| DCIS is not deadly and the risks of trial does not outweigh benefit | Comments from participants indicating that they feel the risk of participating in a clinical trial for DCIS is not worth it because DCIS is not deadly, is not cancerous, and/or is curable with current SOC surgical approaches. |
| Intervention may not be effective | Comments from participants indicating that they feel the risk of participating in a clinical trial for DCIS is not worth it because the intervention may not effectively treat DCIS or prevent future DCIS recurrence. |
| Unknown side effects | Comments from participants indicating that they feel the risk of participating in a clinical trial for DCIS is not worth it because the intervention may cause side effects that are not yet known. |
| Potential to cause harm to participant | Comments from participants indicating that they feel the risk of participating in a clinical trial for DCIS is not worth it because the intervention may cause harm or hurt them. |
| May receive placebo | Comments from participants indicating that they feel the risk of participating in a clinical trial for DCIS is not worth it because they may receive placebo or no treatment at all. |
| Time commitment | Comments from participants indicating that they feel the risk of participating in a clinical trial for DCIS is not worth it because it would take a significant amount of time (additional testing, taking surveys, additional exams, etc.) |
| Travel requirements | Comments from participants indicating that they feel the risk of participating in a clinical trial for DCIS is not worth it because it would require additional travel efforts such as coordinating transportation and/or lodging, time of travel, cost of travel, etc. (may overlap with Time Commitment above or Financial Losses below) |
| Financial Losses | Comments from participants indicating that they feel the risk of participating in a clinical trial for DCIS is not worth it because it would require additional costs including cost of transportation, time lost to time off from work, lodging costs, etc. |
| Distrust / Historically unjust research practices | Comments from participants indicating that they feel the risk of participating in a clinical trial for DCIS is not worth it because of their distrust of medical research and/or healthcare at large due to medicine’s unjust medical and research practices especially affecting marginalized communities (the black and African American communities, the disabled community, etc.) |
| Unable to be adherent to research regimen | Comments from participants indicating that they feel the risk of participating in a clinical trial for DCIS is not worth it because the clinical trial would require great effort and/or they would be unable to keep up with the research regimen |
| Delay standard of care treatment | Comments from participants indicating that they feel the risk of participating in a clinical trial for DCIS is not worth it because it would delay them receiving standard of care therapy for DCIS. |
| Financial Incentive | Comments from participants indicating that they feel the risk of participating in a clinical trial for DCIS is not worth it because of interpretation that financial reward for participation indicates a riskier trial. |
| Other | Comments from participants indicating that they feel the risk of participating in a clinical trial for DCIS is not worth it because of a reason that is not specified above. |
| Ambivalence Regarding Participating in Clinical Trials specifically for DCIS | Comments from participants indicating do not feel strongly positively or negatively toward their willingness to participate in a DCIS clinical trial. |
| Ambivalence Present | Comments from participants indicating do not feel strongly positively or negatively toward their willingness to participate in a DCIS clinical trial. This may include statements that specify the needed conditions or qualifiers for them to choose to participate in a trial for DCIS or not. |
| Positive Influential Factors for Participating in Clinical Trials (in general)IN GENERAL | Comments from participants indicating their willingness to participate in clinical trials in general. |
| Access to newer or better treatments | Comments from participants indicating that the prospect of newer or better treatments potentially offered via clinical trials is desirable and/or would positively influence their participation in a DCIS clinical trial. |
| Increased monitoring by healthcare team | Comments from participants indicating that the prospect of increased monitoring by a healthcare team potentially offered via clinical trials is desirable and/or would positively influence their participation in a clinical trial. |
| Financial incentive | Comments from participants indicating that financial incentive potentially offered via clinical trials is desirable and/or would positively influence their participation in a clinical trial. Financial incentive may include but is not limited to decreased cost of care, decreased cost of treatment, and direct compensation for participation. |
| Contributing to Science/Research | Comments from participants indicating that the prospect of contributing to science and/or research potentially offered via clinical trials is desirable and/or would positively influence their participation in a clinical trial. |
| Helping patients with the same disease in the future | Comments from participants indicating that the prospect helping future patients with the same condition potentially offered via clinical trials is desirable and/or would positively influence their participation in a clinical trial. |
| All other treatment options exhausted | Comments from participants indicating that the prospect of having an additional line of treatment when all others were exhausted potentially offered via clinical trials is desirable and/or would positively influence their participation in a clinical trial. |
| Other | Comments from participants indicating that the prospect of another influential factor not specifically listed above is potentially offered via clinical trials and is desirable and/or would positively influence their participation in a clinical trial. For example, this may include having a family history relevant to the interest of the clinical trial. |
| Negative Influential Factors for Participating in Clinical Trials in GENERAL | Comments from participants indicating their lack of willingness to participate in clinical trials in general. |
| Intervention may not be effective | Comments from participants indicating that they feel the risk of participating in a clinical trial is not worth it because the intervention may not be an effective to treat or prevent the condition from recurring. |
| Unknown side effects | Comments from participants indicating that they feel the risk of participating in a clinical trial is not worth it because the intervention may cause side effects that are not yet known. |
| Potential to cause harm to participant | Comments from participants indicating that they feel the risk of participating in a clinical trial is not worth it because the intervention may cause harm or hurt them. |
| May receive placebo | Comments from participants indicating that they feel the risk of participating in a clinical trial is not worth it because they may receive placebo or no treatment at all. |
| Time commitment | Comments from participants indicating that they feel the risk of participating in a clinical trial is not worth it because it would take a significant amount of time (additional testing, taking surveys, additional exams, etc.) |
| Travel requirements | Comments from participants indicating that they feel the risk of participating in a clinical trial is not worth it because it would require additional travel efforts such as coordinating transportation and/or lodging, time of travel, cost of travel, etc. (may overlap with Time Commitment above or Financial Losses below) |
| Financial Losses | Comments from participants indicating that they feel the risk of participating in a clinical trial is not worth it because it would require additional costs including cost of transportation, time lost to time off from work, lodging costs, etc. |
| Distrust / Historically unjust research practices | Comments from participants indicating that they feel the risk of participating in a clinical trial is not worth it because of their distrust of medical research and/or healthcare at large due to medicine’s unjust medical and research practices especially exploiting marginalized communities (the black and African American communities, the disabled community, etc.) |
| Unable to be adherent to research regimen | Comments from participants indicating that they feel the risk of participating in a clinical trial is not worth it because the clinical trial would require great effort and/or they would be unable to keep up with the research regimen |
| Delay standard of care treatment | Comments from participants indicating that they feel the risk of participating in a clinical trial is not worth it because it would delay them receiving standard of care therapy for their condition. |
| Financial Incentive | Comments from participants indicating that they feel the risk of participating in a clinical trial is not worth it because of interpretation that financial reward for participation indicates a riskier trial. |
| Other | Comments from participants indicating that they feel the risk of participating in a clinical trial is not worth it because of a reason that is not specified above. |
| Ambivalence Regarding Participating in Clinical Trials IN GENERAL | Comments from participants indicating do not feel strongly positively or negatively toward their willingness to participate in a clinical trial. |
| Ambivalence Present | Comments from participants indicating do not feel strongly positively or negatively toward their willingness to participate in a clinical trial. This may include statements that specify the needed conditions or qualifiers for them to choose to participate in a trial or not. |
| Credible Sources For Learning about Clinical Trials | Comments from participants indicating which resources they feel are credible for learning more about clinical trials in general or specific opportunities to participate in a clinical trial. |
| Healthcare Providers (HCPs) | Comments from participants indicating they would seek or have sought the insight of their primary care providers, specialists, or any other healthcare provider on clinical trials. Healthcare providers utilizing web-based interfacing including “MyChart” to share this information falls under this code. |
| Informational Materials such as flyers, brochures, pamphlets | Comments from participants indicating they would seek or have sought information on clinical trials from informational flyers, brochures, pamphlets. Often these informational materials may be encountered in medical offices. |
| Magazines | Comments from participants indicating they would seek or have sought information on clinical trials from magazines including advertisements and articles. |
| Internet | Comments from participants indicating they would seek or have sought information from the internet to learn more about clinical trials. Examples of where participants may have sought information online  Include Google, informational webpages from medical centers, overseeing medical bodies such as the American Cancer Society, a drug company website, etc. Also includes social media or webpages where patients and/or providers may interface via public (not private like MyChart) online discussion boards and similar platforms. |
| Preferences in Discussing Clinical Trials with HCPs/Patients | Comments from participants indicating how they would like to learn about and discuss clinical trials with their healthcare teams, specifically which setting or mode of communication they would prefer. This code can also be used for healthcare providers participating in the focus groups if they indicate their preference in setting/mode of communication to discuss clinical trials with their patients. |
| In-Person | Comments from participants indicating they feel conversations between patients and healthcare providers regarding specific opportunities for clinical trial participation should or could be done in person in a medical setting. |
| Telehealth (video or telephone) | Comments from participants indicating they feel conversations between patients and healthcare providers regarding specific opportunities for clinical trial participation should or could be done via telehealth (video-chatting or telephone) |
| MyChart | Comments from participants indicating they feel conversations between patients and healthcare providers regarding specific opportunities for clinical trial participation should or could be done via MyChart / online text-based interfacing. |
| Mail | Comments from participants indicating they feel conversations between patients and healthcare providers regarding specific opportunities for clinical trial participation should or could be done by mail. |
| Knowledge of Postmenopausal hormone therapy (PHT) | Comments from participants indicating their understanding, familiarity, and prior experiences surrounding PHT. |
| Present | Comments from participants indicating they know of PHT and/or possess factual knowledge about PHT (mechanism of action, indications for use, side effects, etc.) |
| Not Present | Comments from participants indicating they do not know of PHT and/or do not possess factual knowledge about PHT (mechanism of action, indications for use, side effects, etc.) |
| Perceptions of PHT | Comments from participants indicating their feelings towards PHT |
| Positive Perceptions / Perceived Benefit | Comments from participants indicating they perceive PHT positively and/or indicate their perceived benefits of using PHT |
| Negative Perceptions / Perceived Risk | Comments from participants indicating they perceive PHT negatively and/or indicate their perceived risks of using PHT |
| Neutral | Comments from participants indicating they do not hold positive nor negative perceptions of PHT |
| Likelihood of Using PHT Personally or Recommending to a Friend | Comments from participants indicating their likelihood to use PHT personally and/or to recommend its use to a friend |
| Would Consider | Comments from participants indicating they would consider using PHT personally and/or would recommend its use to a friend (within the context of a clinical trial for DCIS, use as currently FDA approved, etc.) |
| Would Not Consider | Comments from participants indicating they would not consider using PHT personally and/or would not recommend its use to a friend (within the context of a clinical trial for DCIS, use as currently FDA approved, etc.) |
| Feedback on PROMISE study Clinical Trial Recruitment Materials | Comments from participants surrounding the PROMISE Facebook page including the informational video. |
| Preferences and information to Include | Comments from participants indicating the information they feel should be included in the PROMISE study recruitments materials. This code can be used for all statements surrounding positive feedback on the materials. |
| Information not needed or harmful to include | Comments from participants indicating the information they feel should not be included in the PROMISE study recruitment materials. This code can be used for all statements surrounding negative feedback on the materials. |
| General Takeaways for Developing Clinical Trial Recruitment Materials | Comments from participants indicating general preferences in clinical trial recruitment materials not necessarily specific to the PROMISE study materials but can also be helpful for future studies. |
| Information to Include | Comments from participants indicating general preferences in what information they would like to see in clinical trial recruitment materials |
| Ways to Present the Information | Comments from participants indicating how they would like information to be presented regarding clinical trials in recruitment materials (tone, type of delivery, animated vs. delivered by a person, etc.) |
| Healthcare and Clinical Trials in the era of COVID-19 (patients and/ vs. providers) | Comments from participants indicating how COVID-19 has affected (or not affected) healthcare including the ability to have appointments, participate in clinical trials, etc. |
| General Effects of COVID-19 on Healthcare Utilization | Comments from participants indicating their personal healthcare utilization during the pandemic. This code can also be used for healthcare providers discussing how they have seen the pandemic affect their clinical/hospital work. |
| Comfort of in-person visits | Comments from participants indicating their comfort in having in-person visits. |
| Comfort of telehealth visits | Comments from participants indicating their comfort in having telehealth visits. |
| Useful digital tools used during pandemic for healthcare | Comments from participants indicating technology they have used during the pandemic to access their healthcare or do things for their health |
| Preference in discussing Clinical Trials during COVID-19 | Comments from participants indicating their comfort and preferences in learning about and discussing clinical trials during the pandemic |
| Barriers to Participation in Clinical Trials during COVID-19 | Comments from participants indicating any barriers to participating in clinical trials they have experienced, seen, or predict from the pandemic. |

**Supplemental Figure 1**

Screenshot of the Promise Study Facebook Webpage


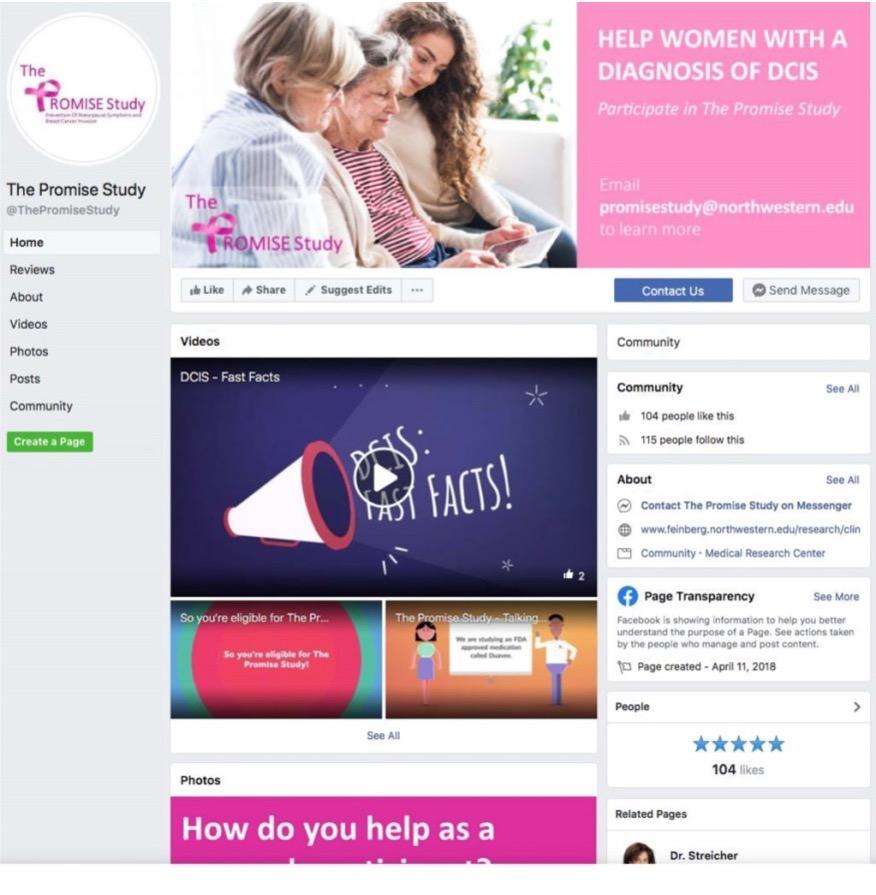


**Supplemental Figure 2**


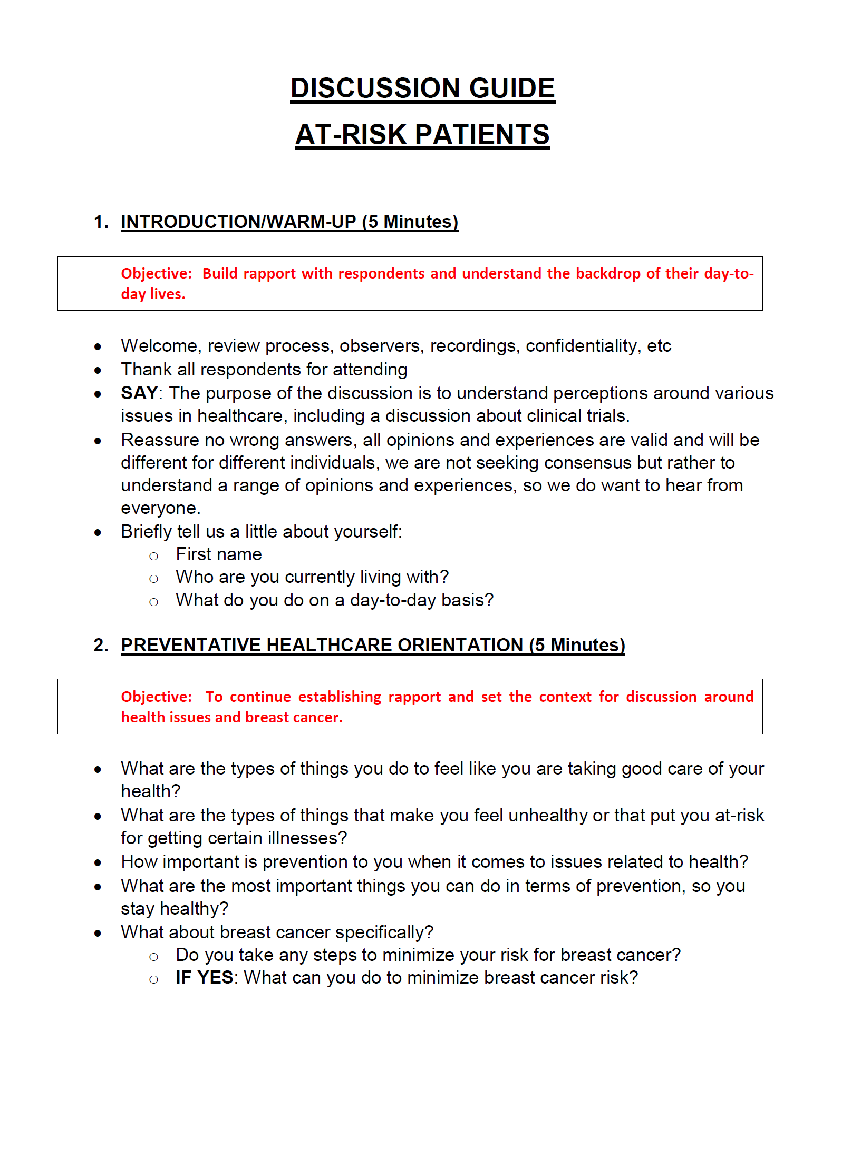

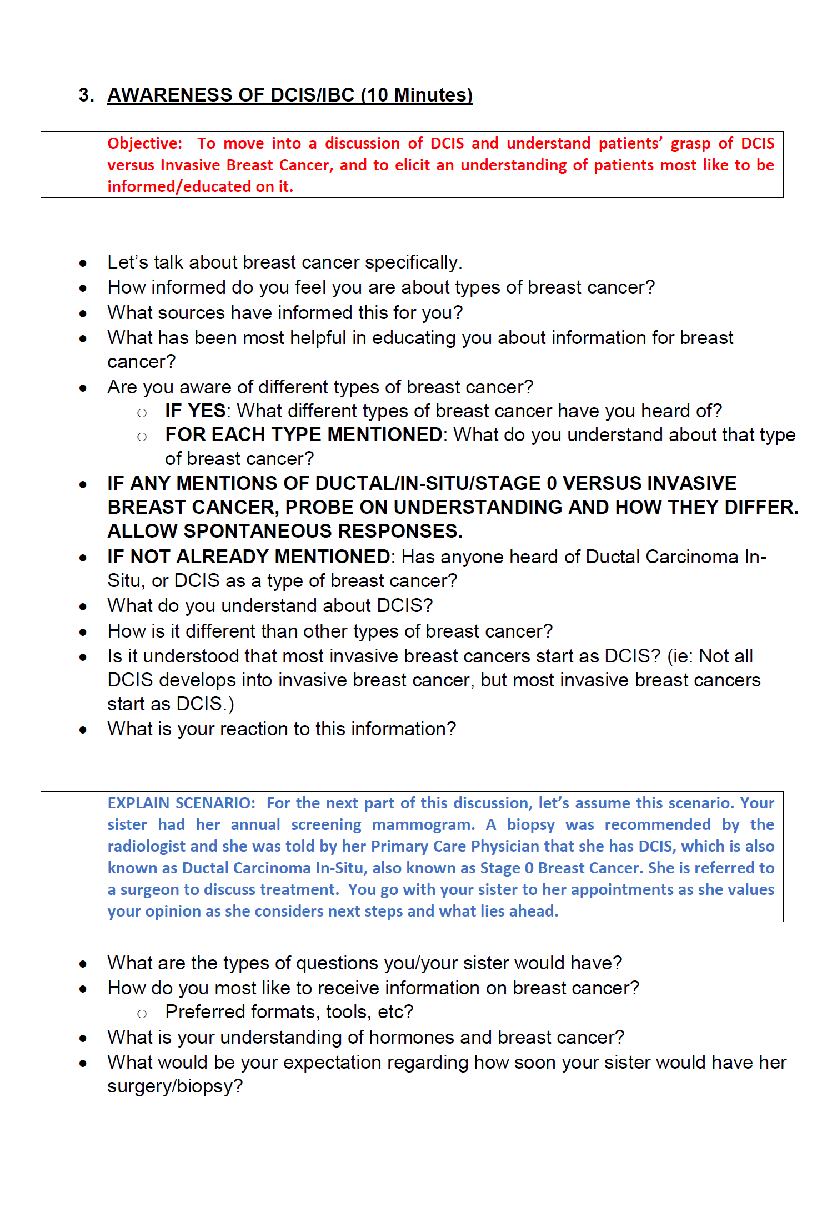


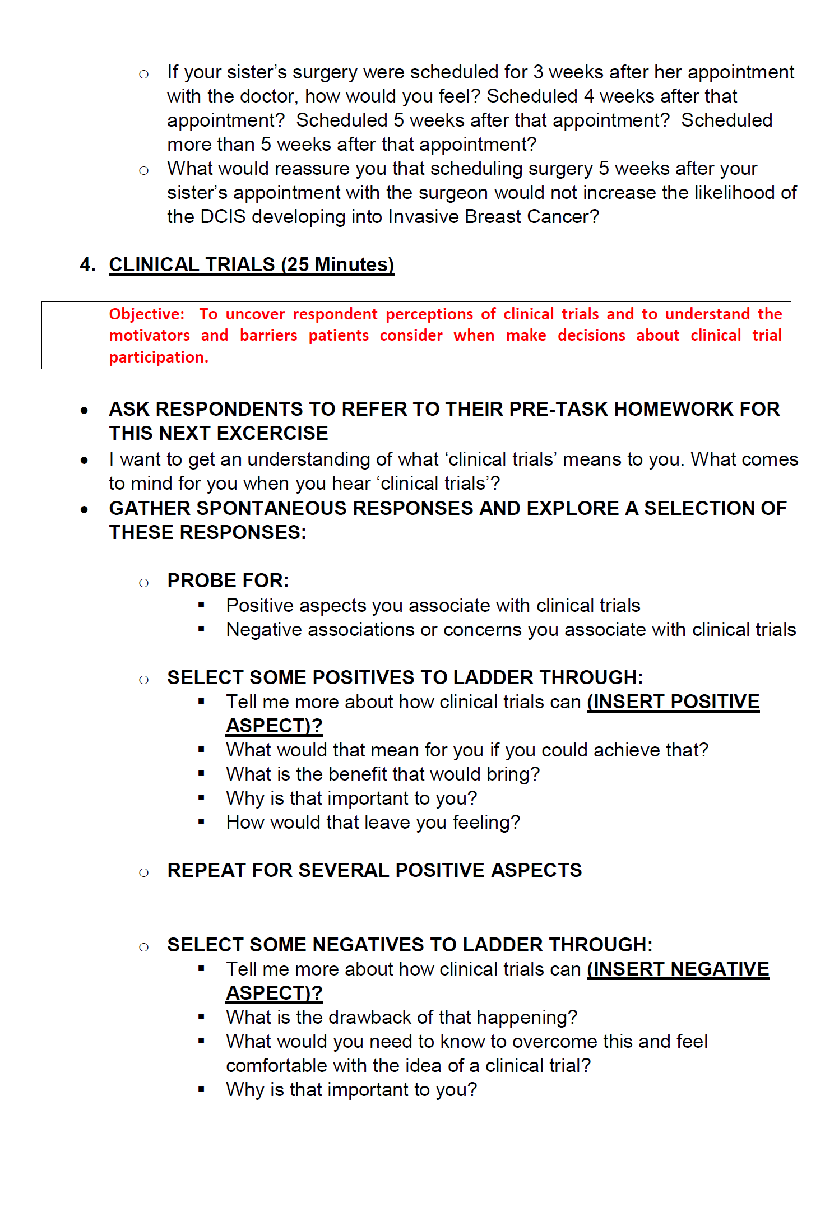


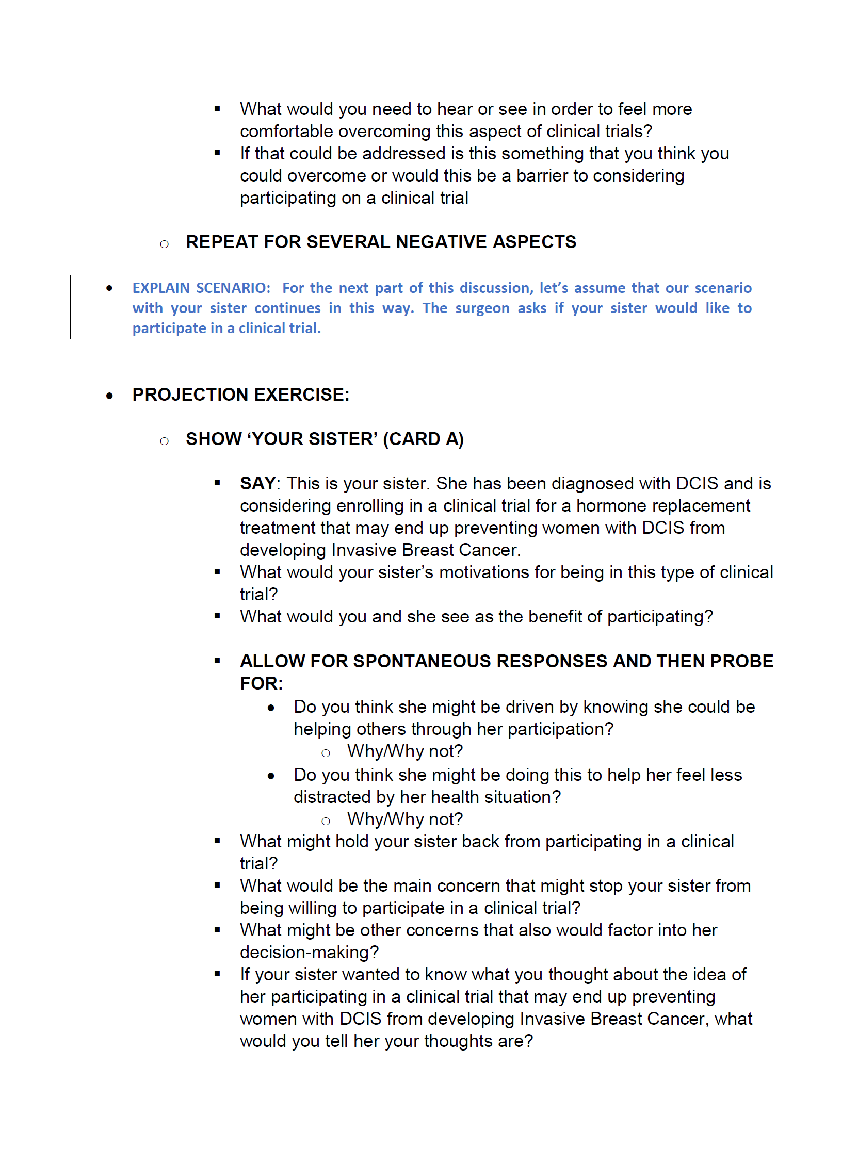


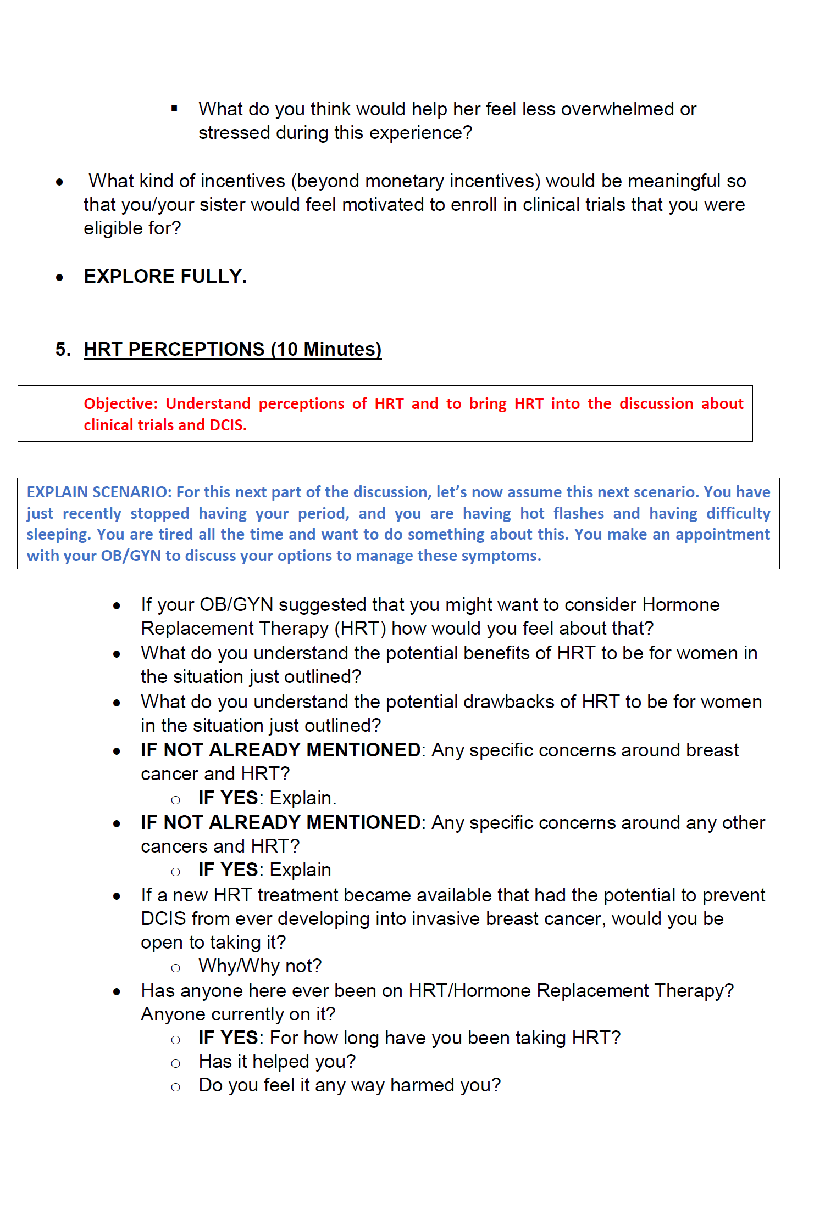


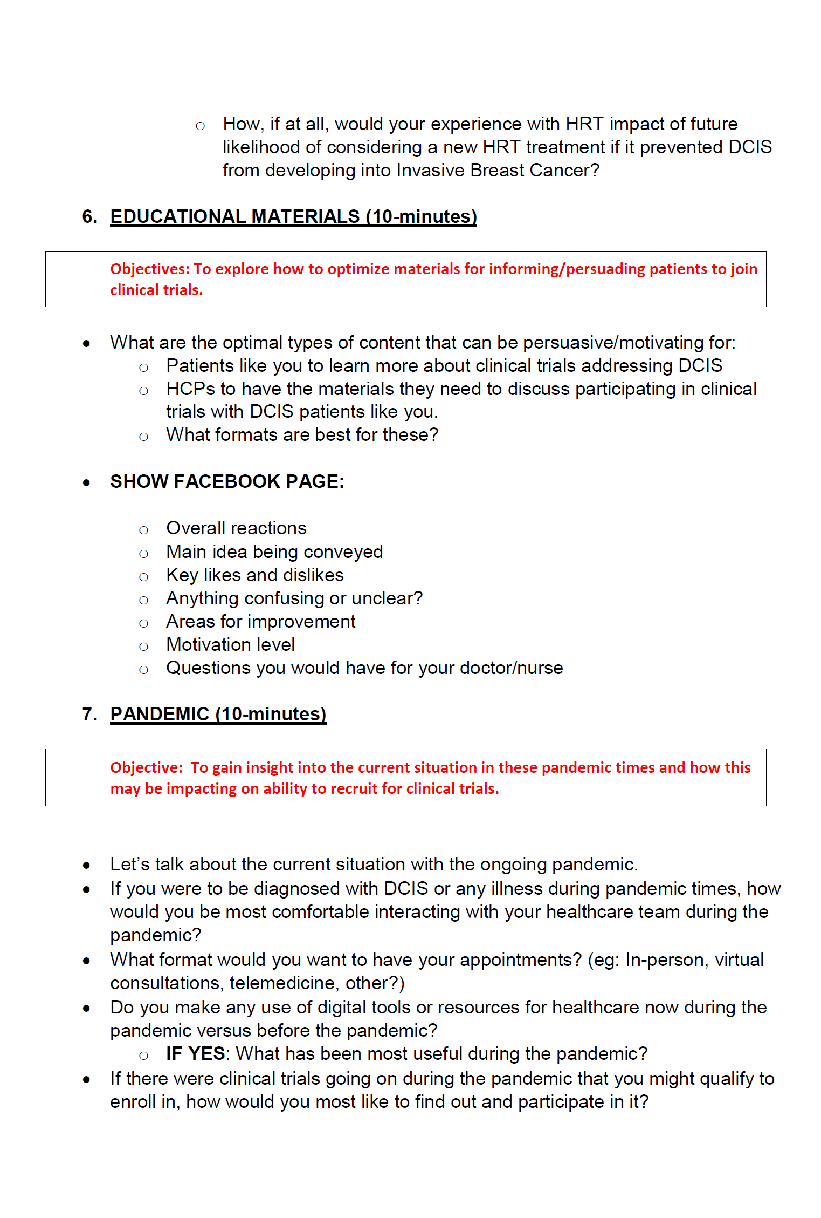


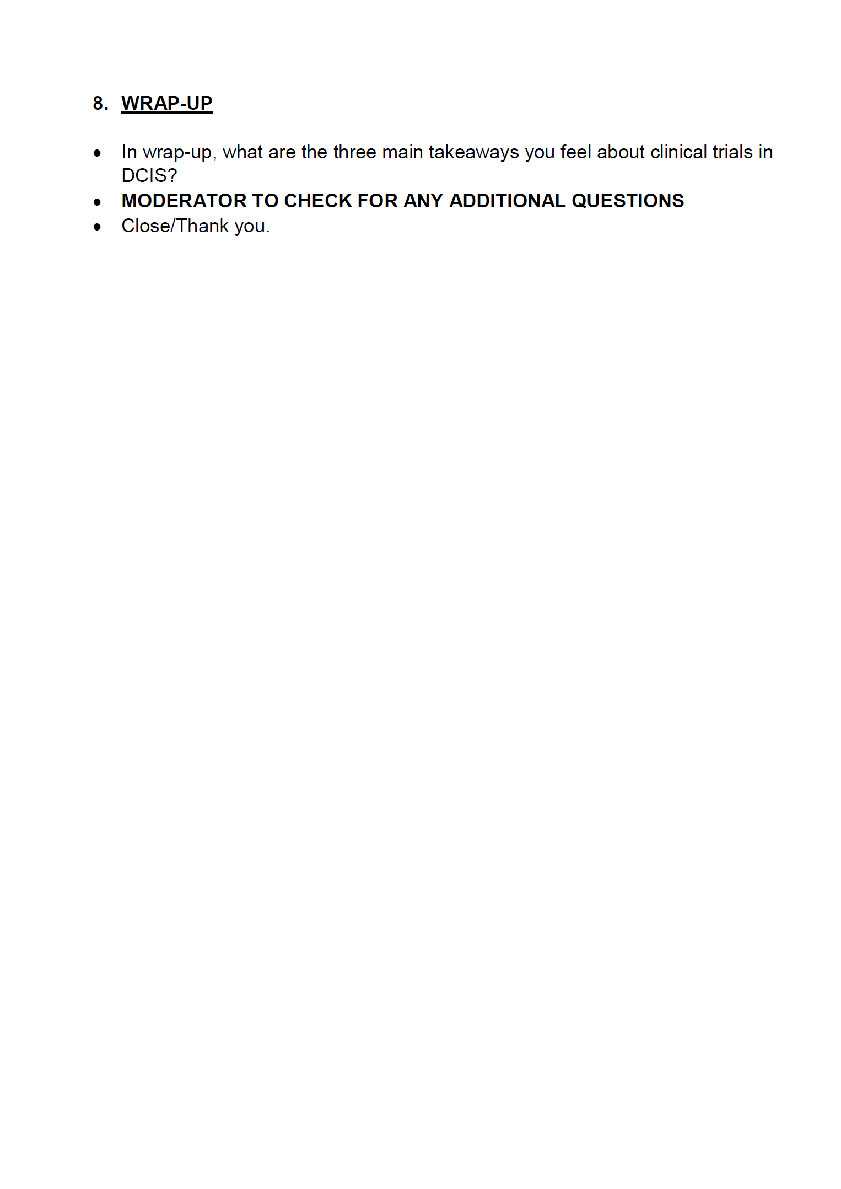


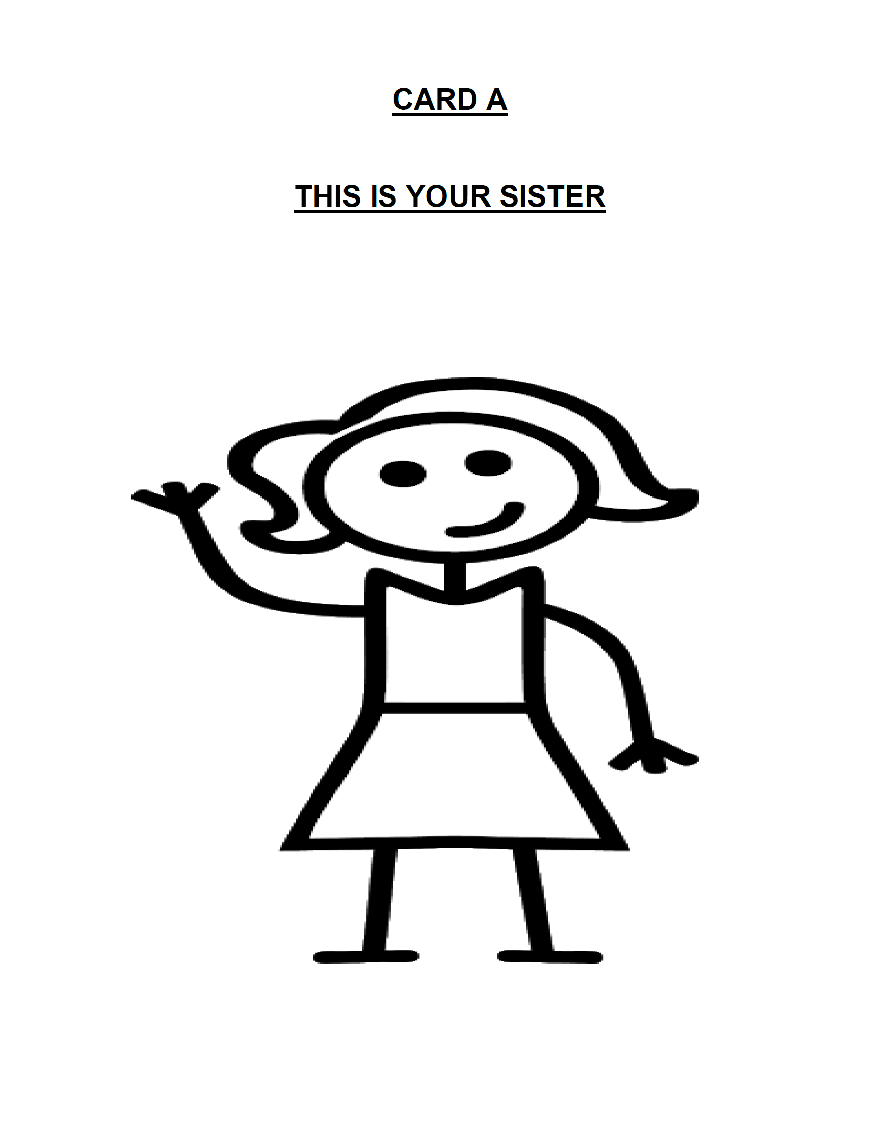


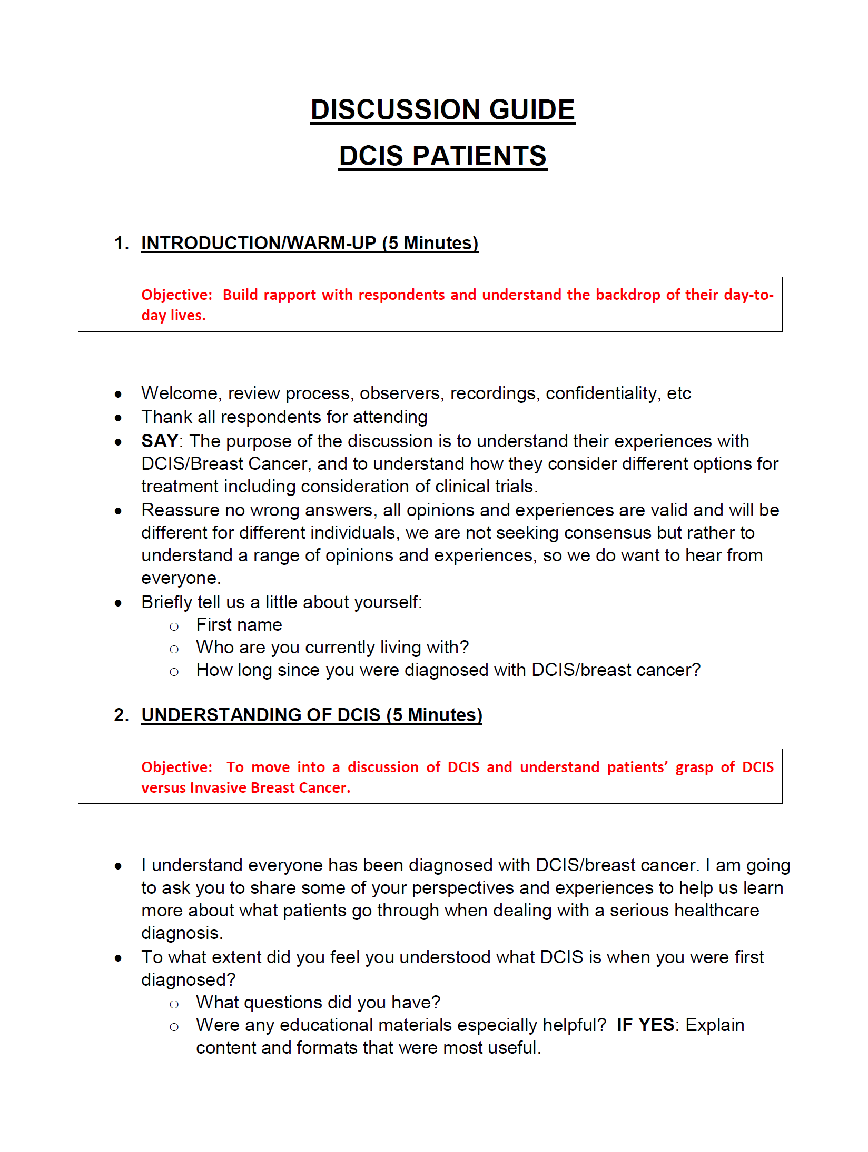


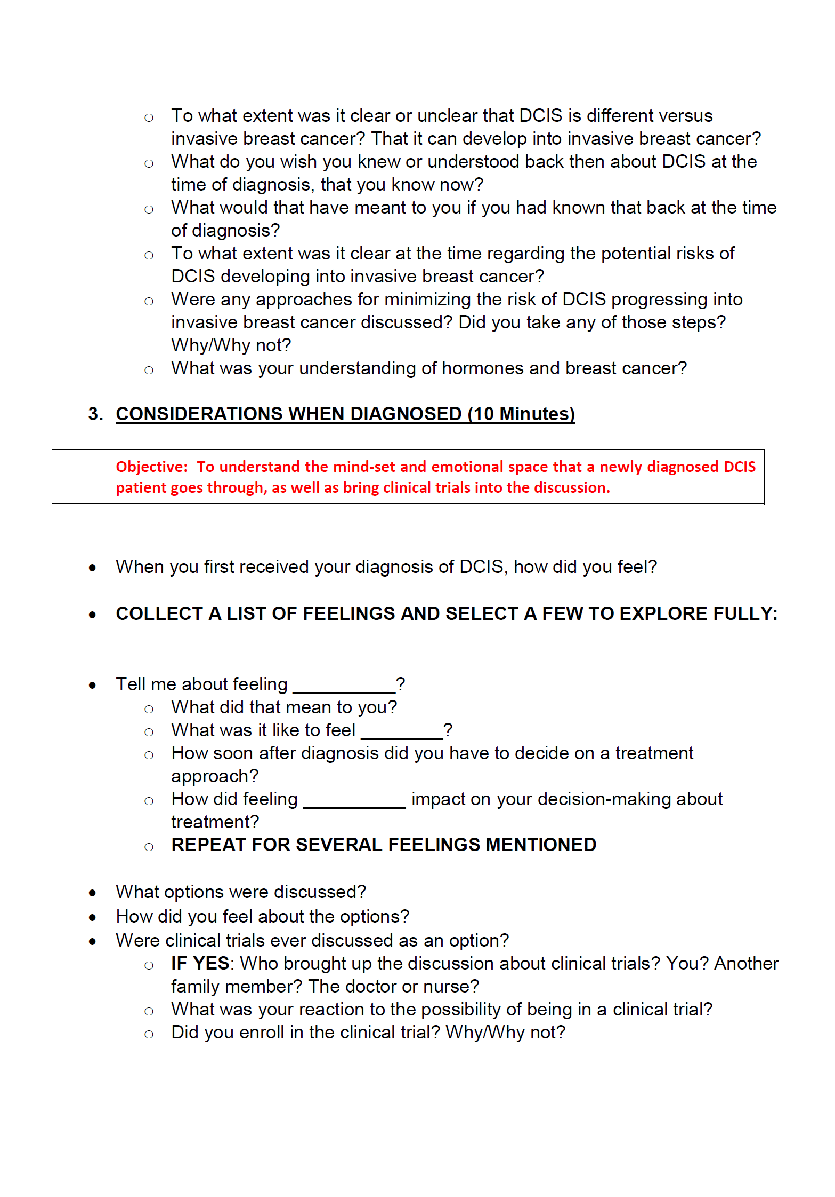


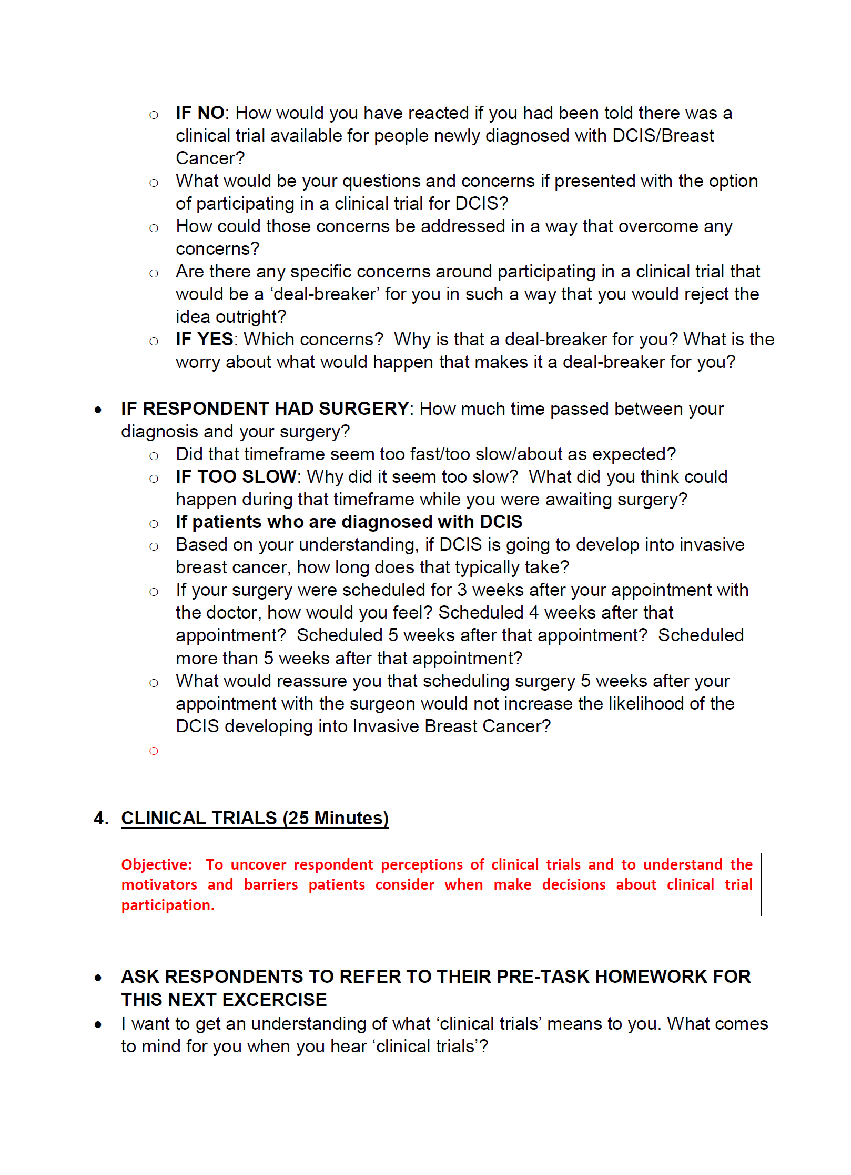


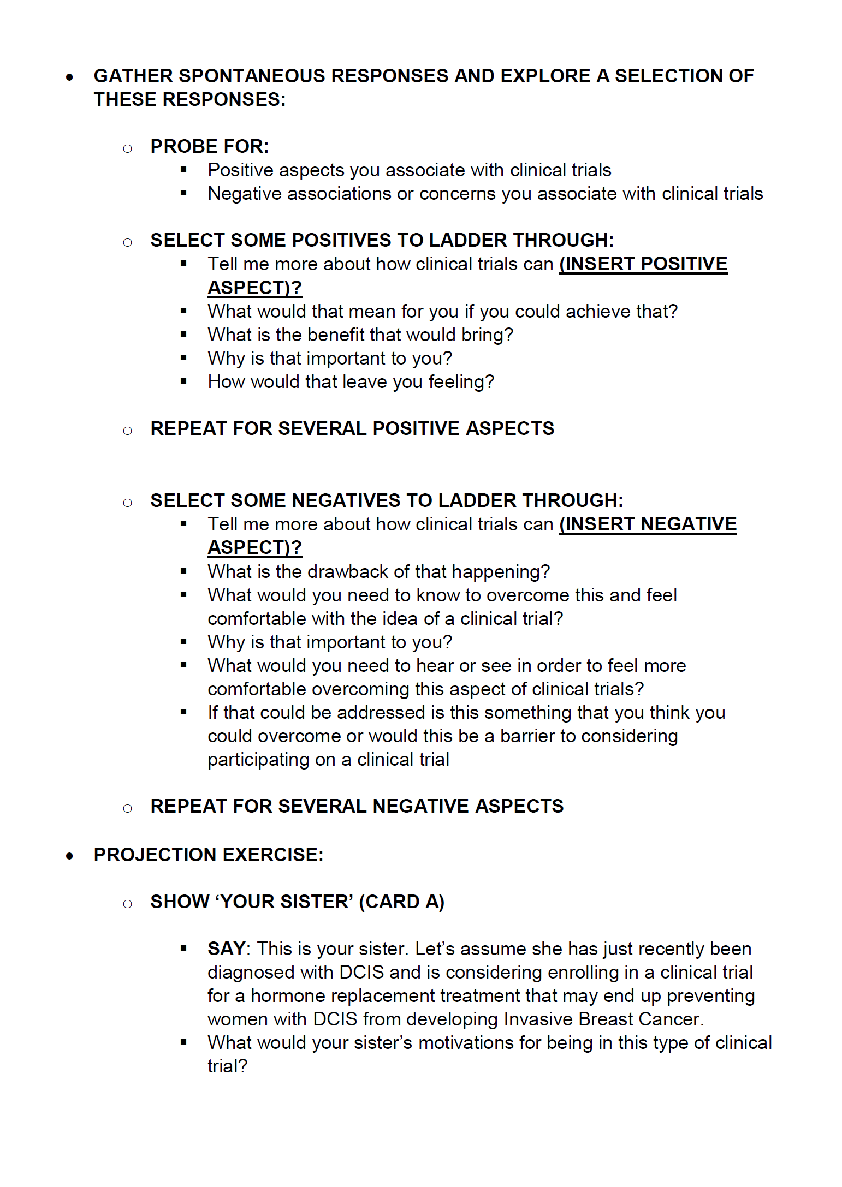


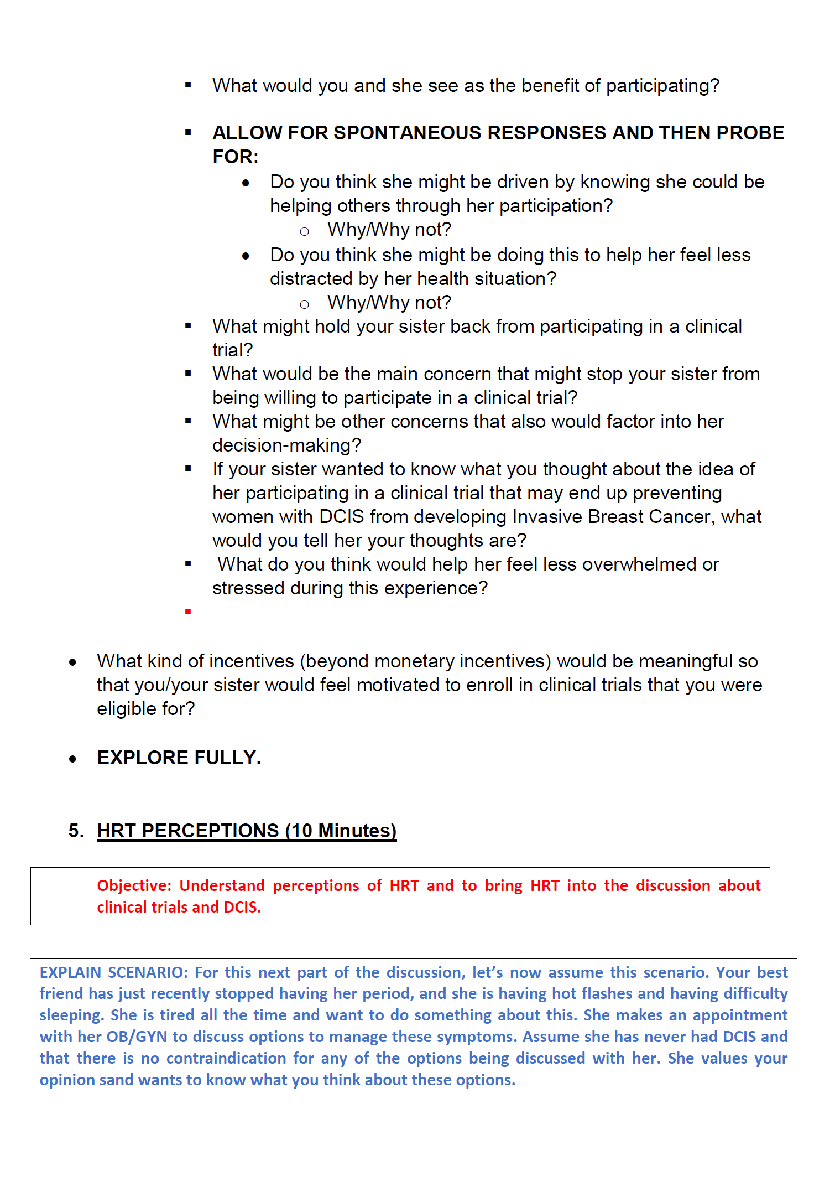


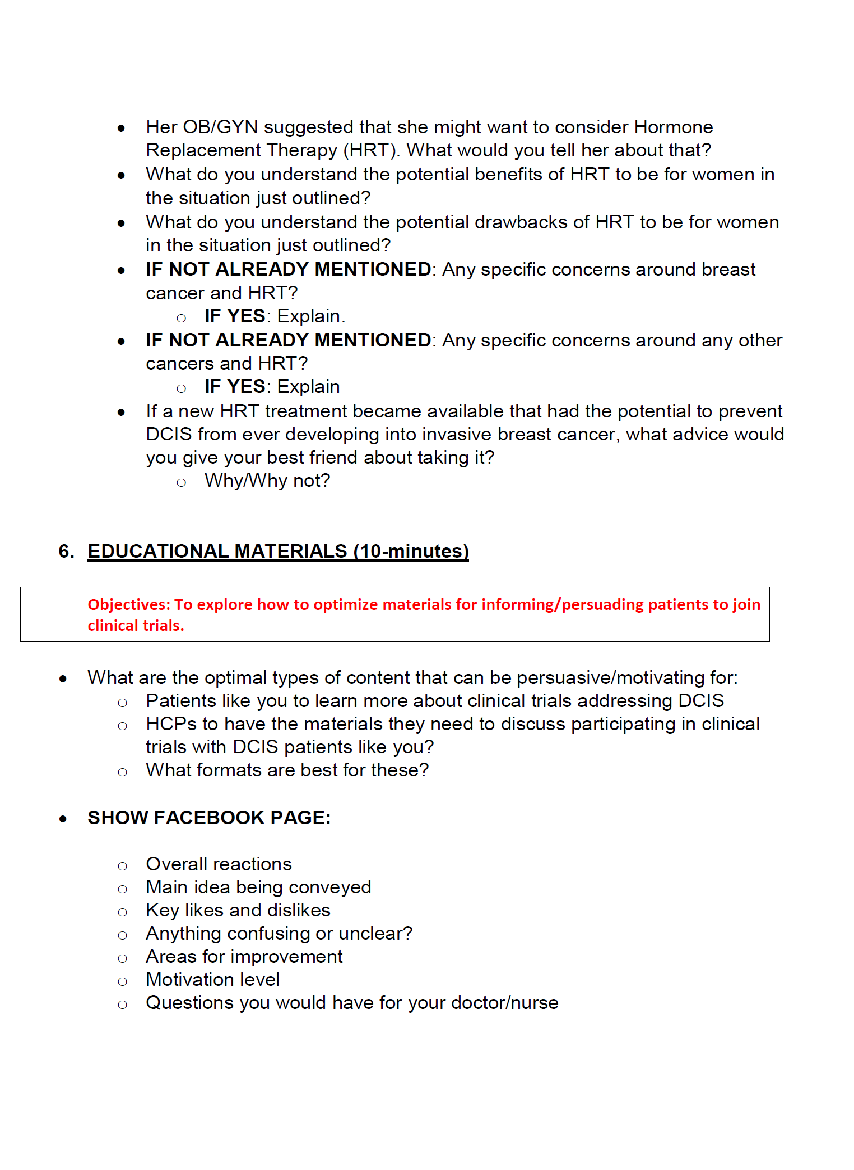


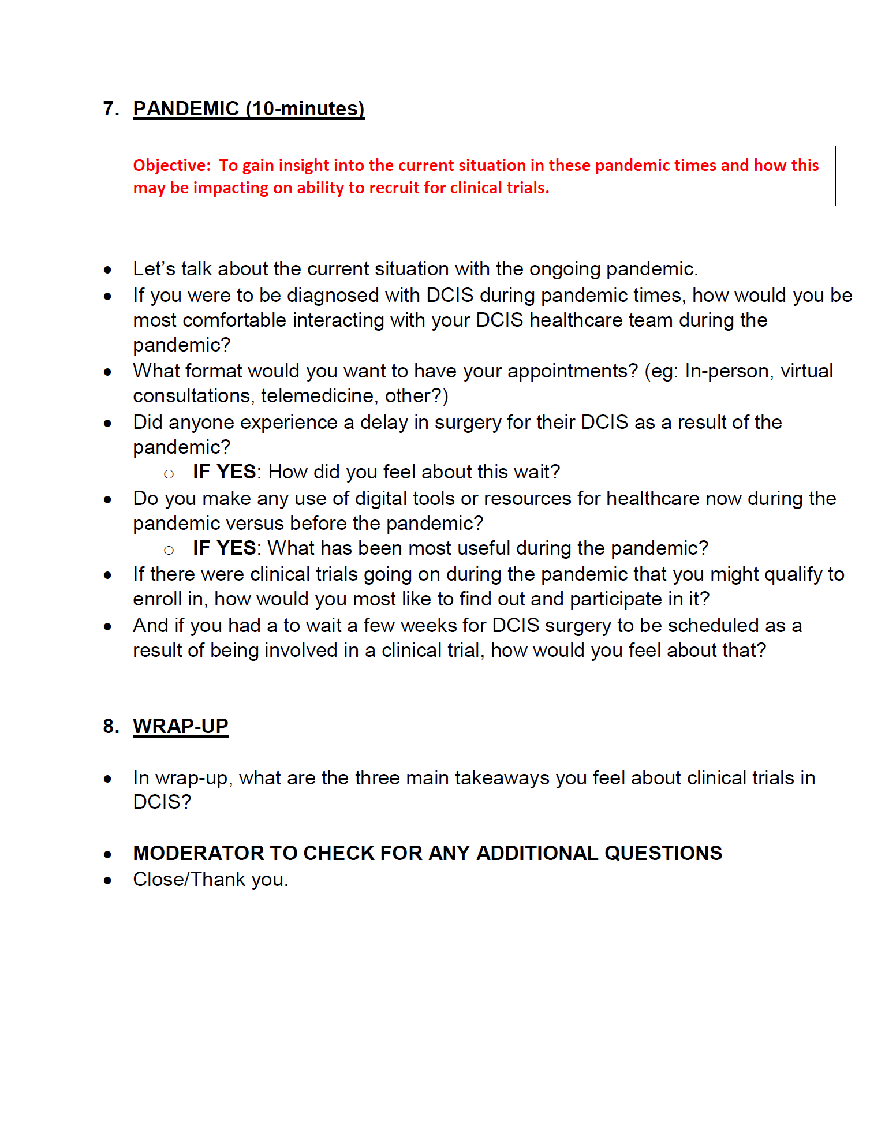


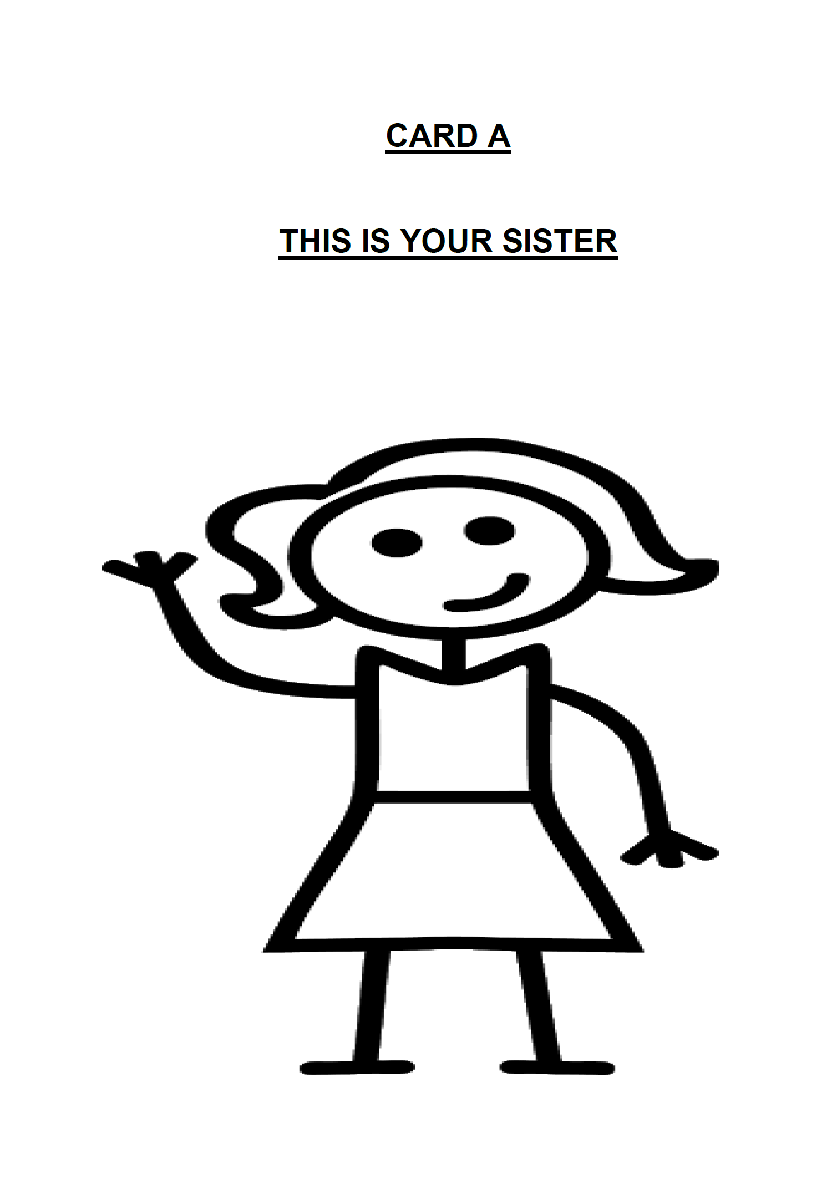


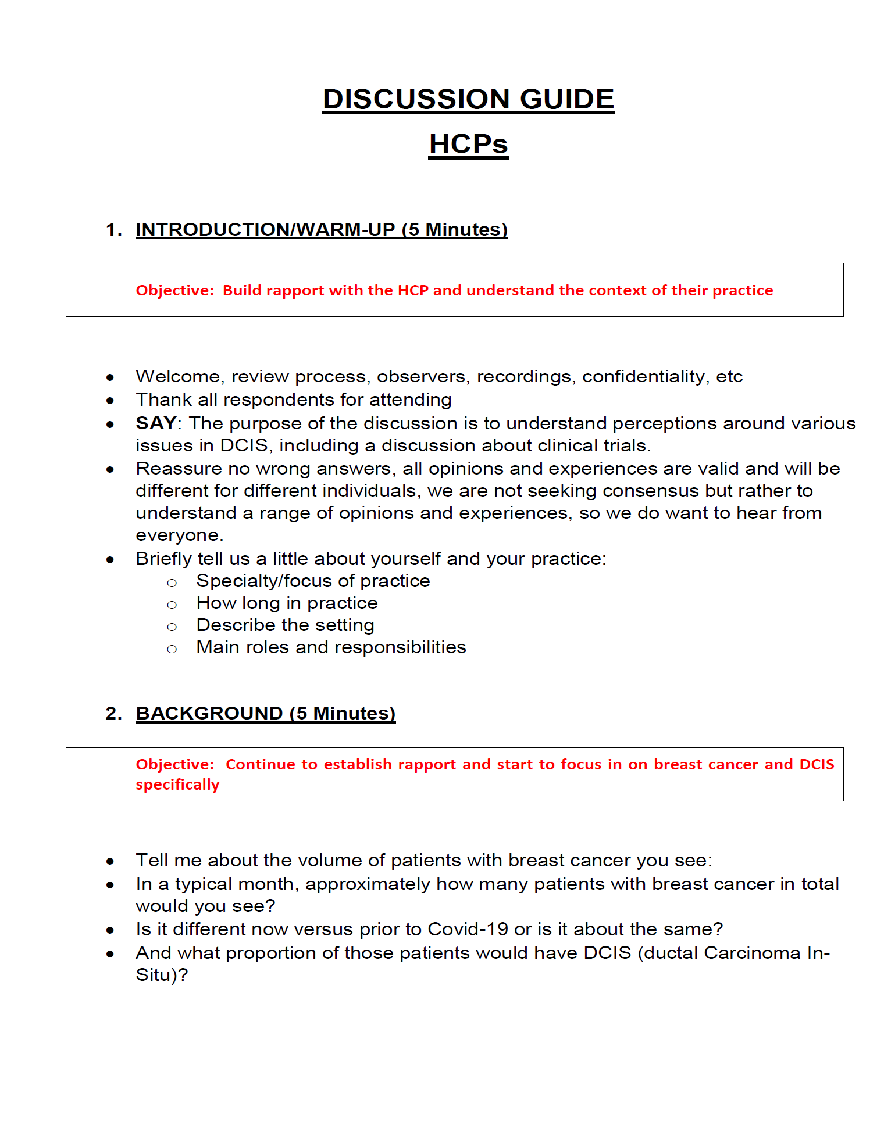


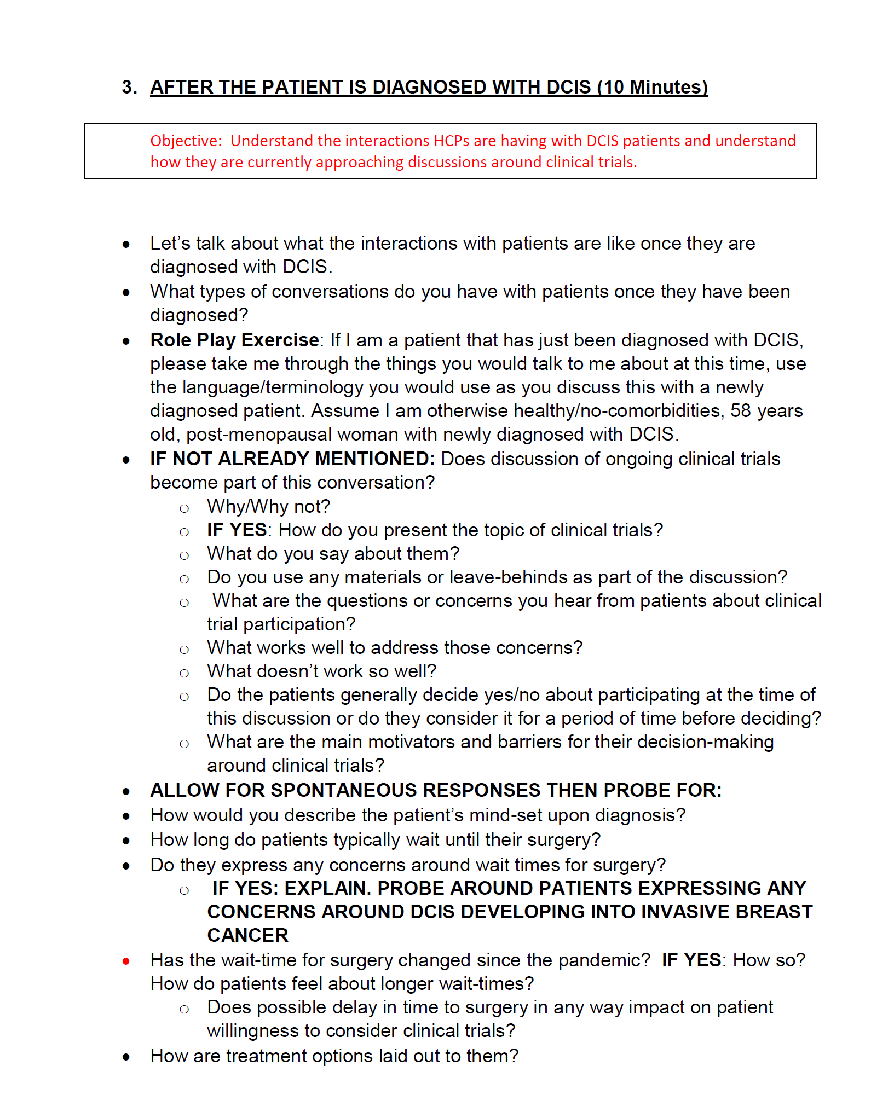


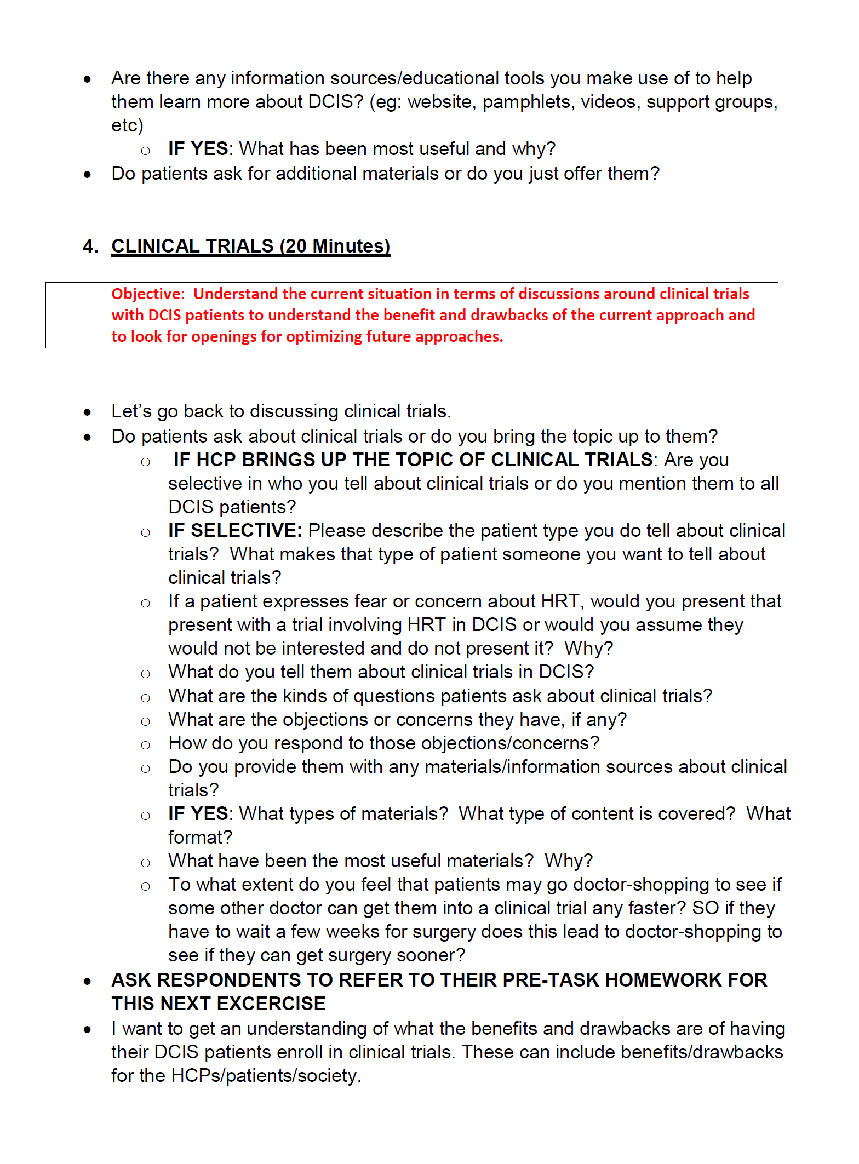


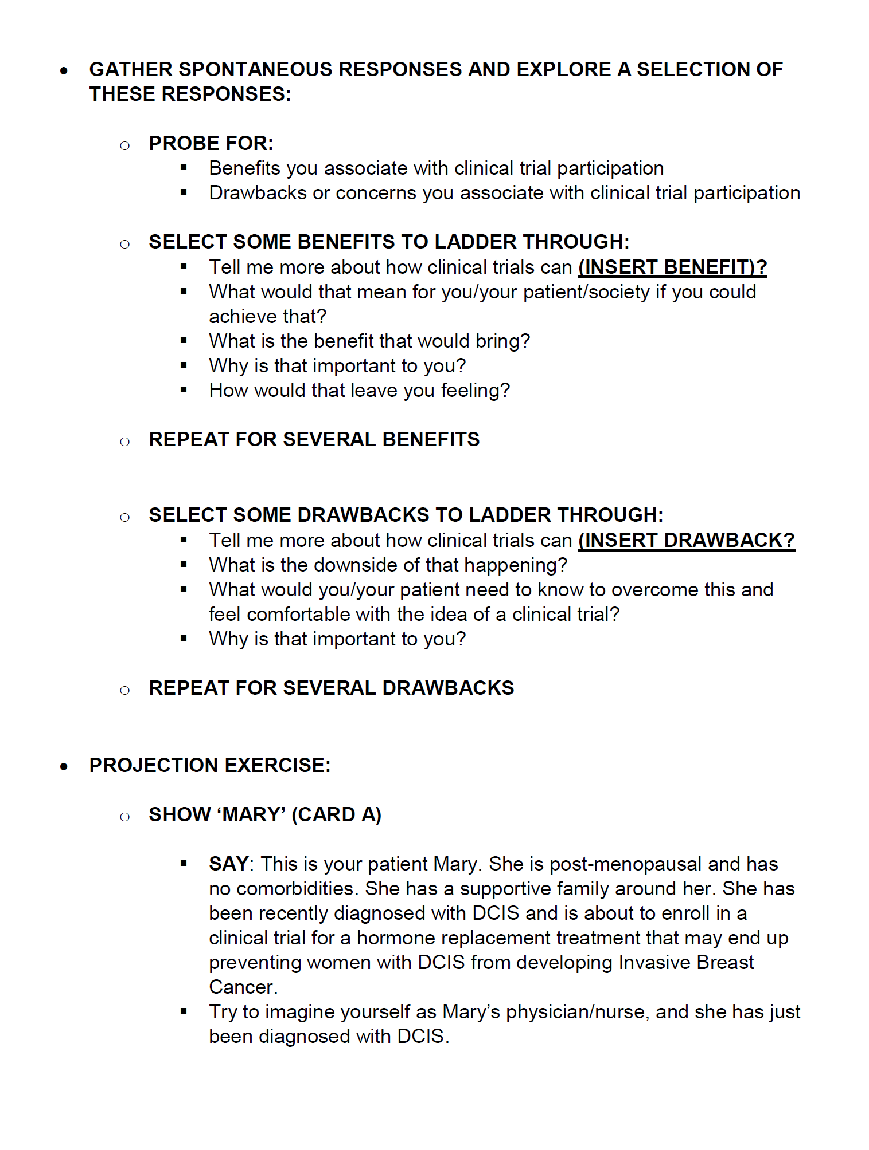


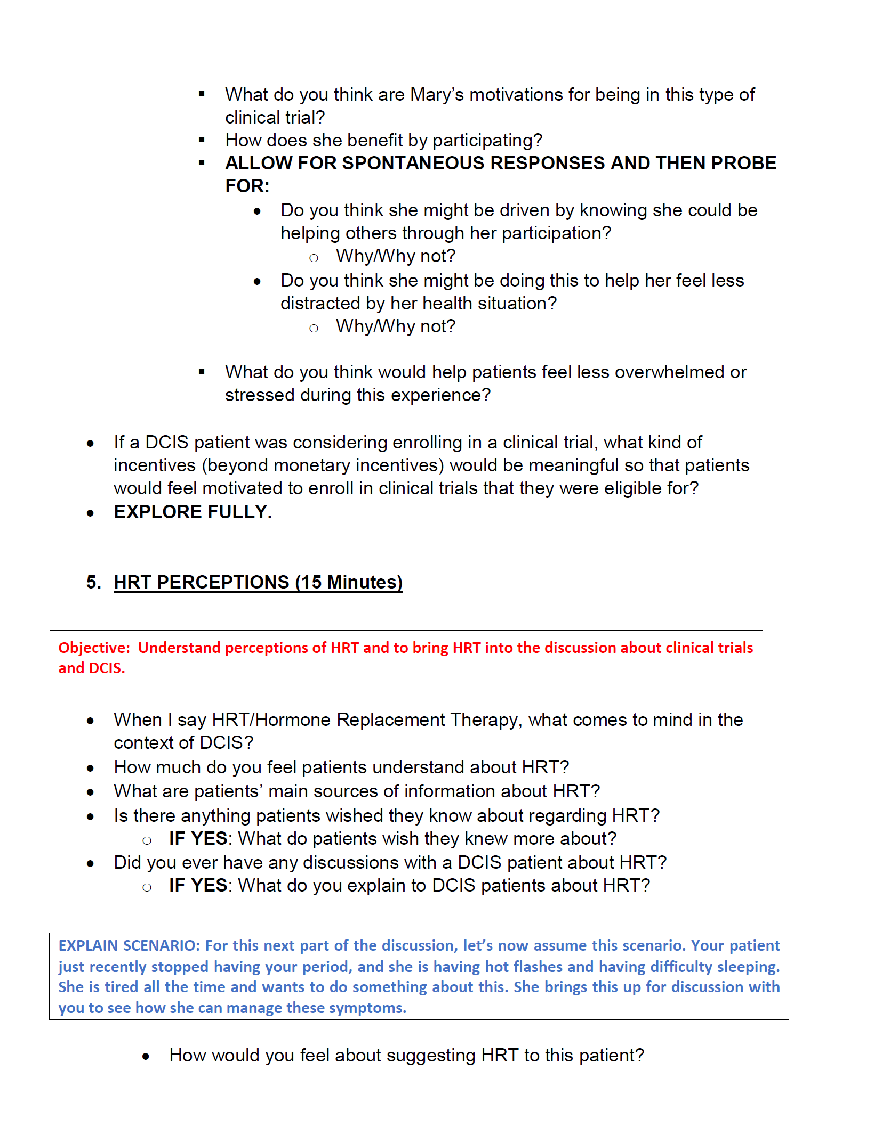


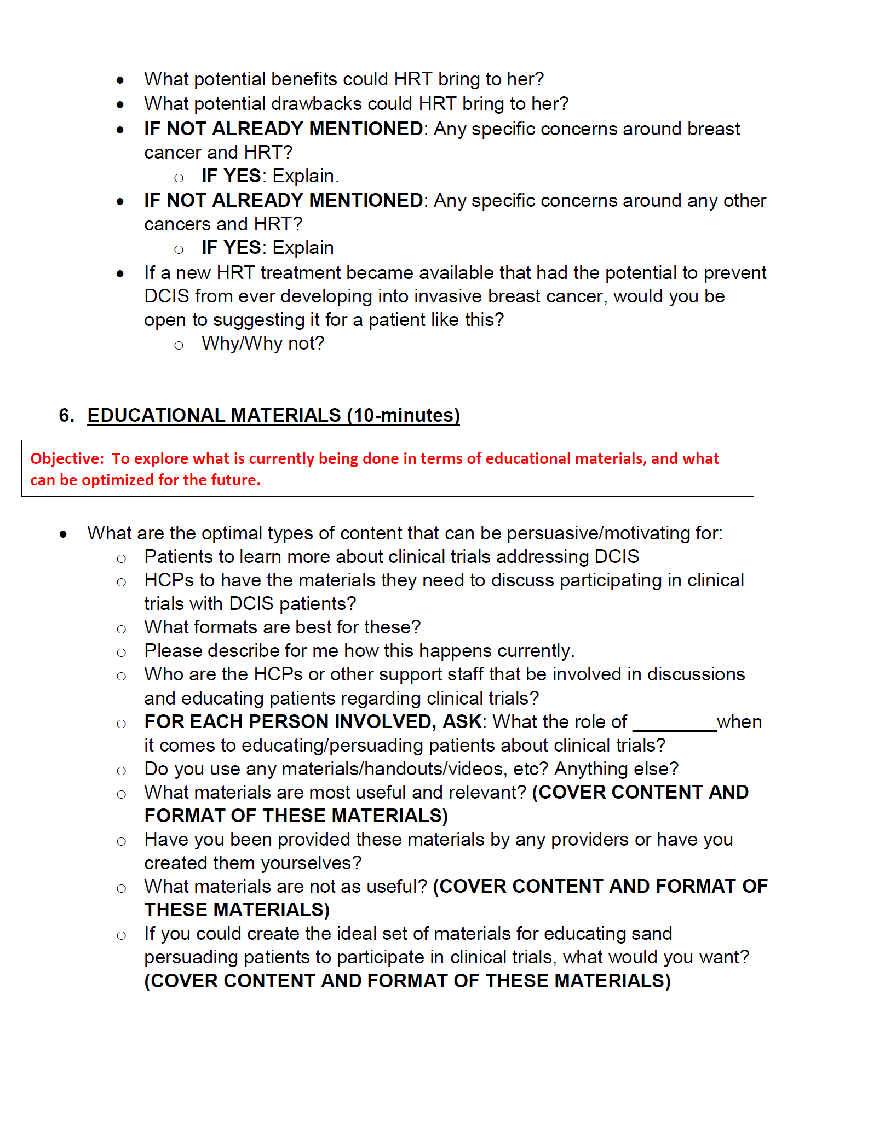


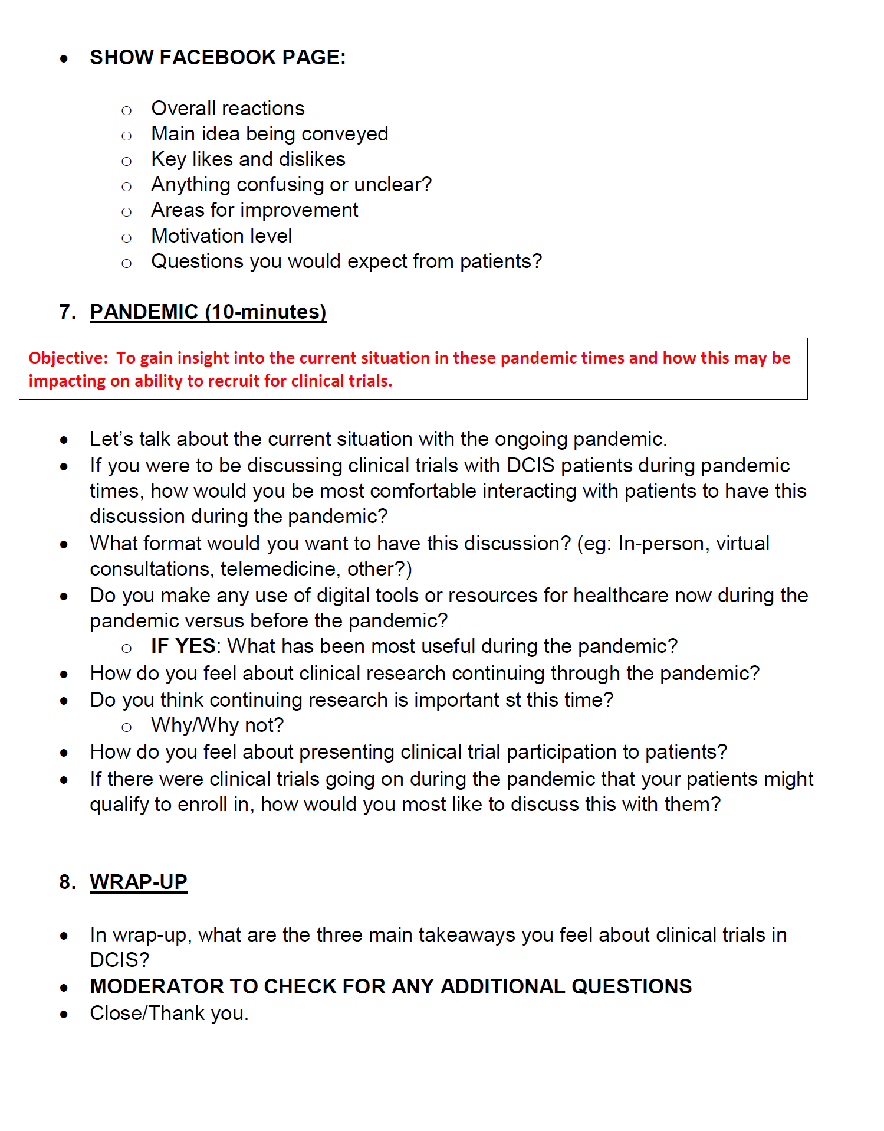


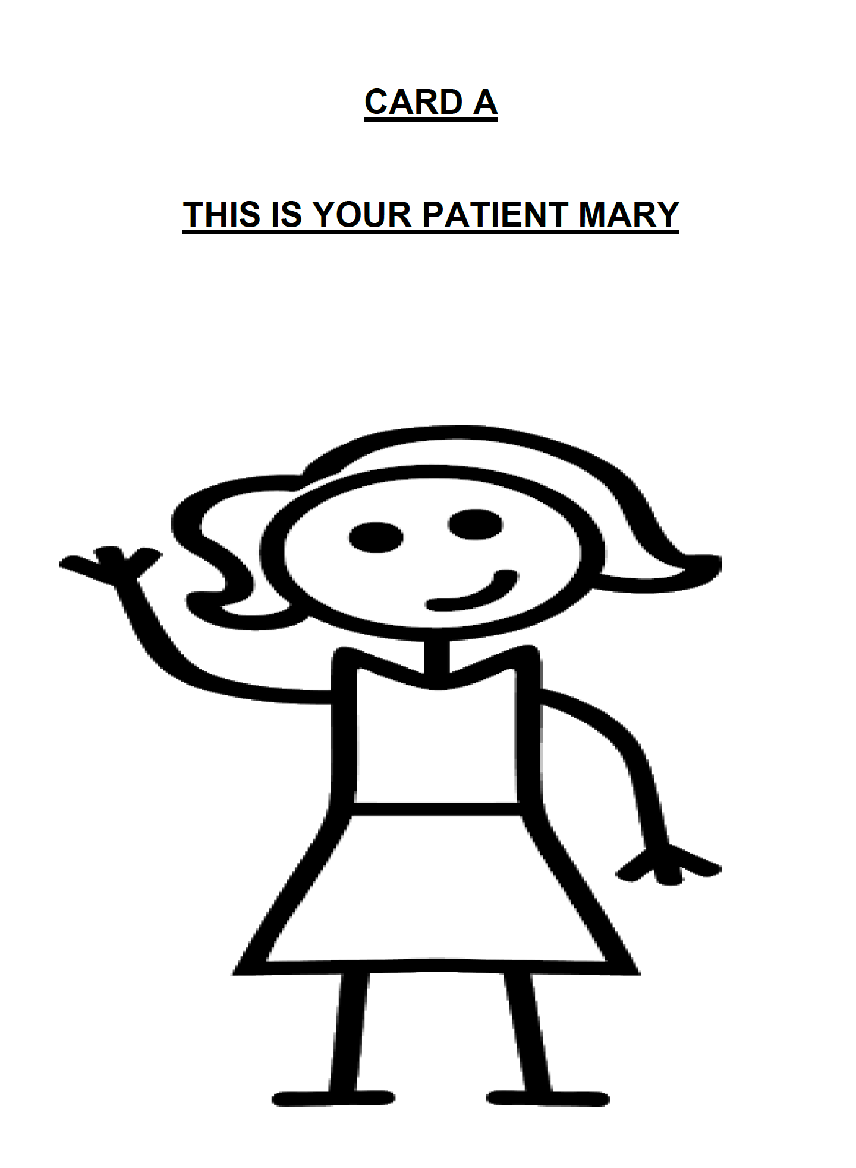

Supplement: Supplementary file 1 — Supplementary file1 (DOCX 3443 KB) [file 10549_2025_7742_MOESM1_ESM.docx]
